# Persistence of a declining anuran species across its distribution

**DOI:** 10.1371/journal.pone.0332991

**Published:** 2025-09-22

**Authors:** Erin L. Koen, E. Hance Ellington, William J. Barichivich, Howard Kochman, Kevin M. Enge, Susan C. Walls

**Affiliations:** 1 Cherokee Nation System Solutions, Contracted to U.S. Geological Survey, Wetland and Aquatic Research Center, Gainesville, Florida, United States of America; 2 Range Cattle Research and Education Center, University of Florida, Ona, Florida, United States of America; 3 Department of Wildlife Ecology and Conservation, University of Florida, Gainesville, Florida, United States of America; 4 U.S. Geological Survey, Wetland and Aquatic Research Center, Gainesville, Florida, United States of America; 5 Fish and Wildlife Research Institute, Florida Fish and Wildlife Conservation Commission, Gainesville, Florida, United States of America; Shiraz University, IRAN, ISLAMIC REPUBLIC OF

## Abstract

Information on a species’ population dynamics, such as changes in abundance and distribution, can be used to identify declining populations and initiate conservation efforts and protections. For the Ornate Chorus Frog (*Pseudacris ornata*), anecdotal observations of local extirpation and population declines have been noted, but trends in its range-wide population status are generally unknown. We used 2227 verified records of Ornate Chorus Frog presence from across the species’ distribution, grouped into 407 populations, and a modified Cormack-Jolly-Seber survival analysis to estimate the probability that historical Ornate Chorus Frog populations persist in the year 2024. Our results suggested that > 36% of historical Ornate Chorus Frog populations are possibly extirpated (probability of persistence < 0.5) and that 33% of populations had a probability of persistence > 0.9. Many of these extant populations occurred in northwestern Florida, southeastern Alabama, and southern Georgia, USA. The probability of persistence was positively influenced by habitat suitability and mean winter precipitation and negatively influenced by urban imperviousness. Ornate Chorus Frogs in protected areas had a higher average probability of persistence compared to populations that were not in protected areas. Our study fills a knowledge gap by identifying regions where Ornate Chorus Frog populations are likely thriving and regions where they may be extinct.

## 1. Introduction

The last 100 years have been defined by rates of biodiversity loss unprecedented in human history [1,2], suggesting that we may be approaching the Earth’s sixth mass extinction [3,4]. While extinction rates have been increasing for most taxonomic groups assessed [5–7], amphibian extinctions have been disproportionately large [1,8,9]. In fact, Daszak et al. [10] posit that the global decline in amphibian populations is one of the most pressing environmental problems in recent times. Globally, over 40% of amphibian species are threatened with extinction [11–13]. An additional 25% of amphibian species are classified as data-deficient [14], meaning that we do not have enough scientific information to evaluate their current conservation status. Thus, estimates of the number of amphibian species currently at risk of extinction are likely much higher than currently known [8], and could be as much as 50% higher or more [14,15].

Causes of amphibian declines include the compounding impacts of habitat loss, climate change, pollution, over-exploitation, invasive species, and disease [3,16–20], although the processes are complex [21,22] and continent-scale drivers remain elusive [23]. For many amphibian species, particularly those 25% deemed data-deficient, neither their population trajectory nor the threats to their past or future persistence are well understood. Further, many amphibian species are sensitive to the impacts of climate change, including changes to hydroperiod and phenology—threats that are predicted to increasingly impact amphibian populations in the future [13,24]. Data-deficient amphibian species may even be experiencing undocumented range loss, and many have seemingly been overlooked and thus remain both under-studied and unprotected [25]. Without knowledge of recent population trends, range contraction, or the causes of such changes, it is challenging to initiate conservation efforts and protections.

The Ornate Chorus Frog (*Pseudacris ornata*) is among the most intrinsically sensitive of anuran species in North America due to its geographic rarity and climate specificity [25]. This species is a small, winter-breeding anuran that is native to the coastal plains of seven states in the Southeast USA (North Carolina (NC), South Carolina (SC), Georgia (GA), Florida (FL), Alabama (AL), Mississippi (MI), and Louisiana (LA)). Until the early 2000s, the Ornate Chorus Frog appeared common and abundant throughout much of its range [26]. Anecdotal observations of population decline and local extirpation have been noted at the range margins, particularly in Louisiana, North Carolina, and peninsular Florida [26,27]. Ornate Chorus Frog populations are considered stable in GA and AL (S5), vulnerable in SC (S3S4), imperiled in FL (S2S3) and NC (S2), critically imperiled in MS (S1), and possibly extirpated in LA (SH) [28,29]. Across their distribution, Ornate Chorus Frogs are threatened by large-scale habitat disturbance and degradation, fragmentation of remaining habitat, disease, and the impacts of climate change, including drought [26].

The life history of the Ornate Chorus Frog makes population monitoring challenging. During the non-breeding period (~May‒Oct), adults spend much of their time burrowed under sand and vegetation, which makes them difficult to detect [30]. During the breeding season (~Nov‒Apr), call surveys can be used to detect vocalizing males, dipnet surveys and environmental DNA sampling of wetlands can detect the presence of tadpoles, and terrestrial drift fences with traps can capture adults and metamorphs as they migrate to or from breeding ponds [26,27]. These methods, however, are labor intensive and difficult to implement at a landscape scale. As a result, range-wide population status and causes of population decline for the species are generally unknown [26].

Despite apparently declining populations across much of its distribution [26,28], we are not aware of any range-wide assessments of Ornate Chorus Frog population status. Assessments of species status are imperative to identify which species are at risk and why—information that could guide conservation and management efforts to halt or reverse declining populations trends. This is particularly true for species like the Ornate Chorus Frog that are sensitive to changing environmental conditions. Community science databases facilitate sharing of species observations across large areas, which can be especially valuable for rare or declining species. Together with emerging modeling techniques that can integrate presence records from a variety of sources [e.g., 31–34], there are new opportunities to assess range-wide population status for species that are otherwise data-limited. Following the methods of Crawford et al. [31], we compiled records of Ornate Chorus Frog presence from various sources, including museums and community science databases, and used a modified Cormack-Jolly-Seber survival analysis to estimate the probability that Ornate Chorus Frog populations across their historical distribution persist.

## 2. Methods

### 2.1. Life history

The Ornate Chorus Frog is a small (35‒40 mm long), terrestrial, winter-breeding amphibian, with life-history traits similar to many of the North American chorus frog species [28]. During the non-breeding season, adult Ornate Chorus Frogs burrow under woody debris near the roots of herbaceous ground vegetation in sandy soils [26,30]. Beginning in the fall (~November), typically after the first heavy autumn rainfalls of the season, adult Ornate Chorus Frogs move from their dry, upland burrows to nearby wetlands and flooded areas (e.g., ditches, fields, ponds) to breed [28,35], preferring relatively small ephemeral wetlands (typically < 1 ha on average, but can be as large as 14 ha; [27,36]) with emergent vegetation that are surrounded by forests with herbaceous ground cover [37]. Arrival time of adults to the breeding ponds varies depending on rainfall patterns but typically peaks in January‒February [35,38]. Males tend to arrive at suitable breeding ponds before gravid females and perch on emergent vegetation or woody detritus to vocalize [38]. Eggs hatch within ~7 days of being deposited and have a relatively short larval period, with metamorphs emerging ~90 days after hatching [28]. By March or April, most adults and metamorphs have left the wetlands and emigrated back to dry, upland habitat [38], usually within 450 m of the breeding pond [27,30,39].

Ornate Chorus Frogs depend on fishless ephemeral wetlands for breeding, making their populations particularly sensitive to environmental conditions that impact hydroperiod. Ornate Chorus Frogs may breed in large wetlands if the wetland had been dry the previous year, as dry-downs lower the likelihood of predator presence (ephemeral ponds are less likely to have fish) [40]. Likewise, Ornate Chorus Frogs tend not to migrate to ponds that experienced unusually high summer or autumn rainfall that prevented the usual annual dry-down [38]. Females that do migrate to ponds that did not dry in the previous summer will postpone breeding and may return the following year to breed if conditions are more favorable [38]. Most Ornate Chorus Frogs, however, breed once during their first, and typically only, year of life [38]. As such, prolonged drought that causes ponds to dry before metamorphs emerge or that precludes filling of ephemeral ponds altogether could threaten the persistence of local populations, especially if the drought lasts for multiple consecutive years [26,27]. The population impacts of reproductive failure from winter drought are likely exacerbated by habitat degradation and habitat loss that reduces connectivity and impedes recolonization following local extirpation [26,27]. Indeed, individual Ornate Chorus Frogs do not necessarily return to their natal wetland to breed, and populations benefit from abundant wetlands in close proximity with variable hydroperiods that can buffer the population against stochastic disturbance, such as drought and loss of individual wetlands [27,40]. Drought that impacts hydroperiod can also cause reproductive failure if the pond dries before metamorphosis occurs [41]. At a landscape scale, Ornate Chorus Frogs are typically associated with upland habitats, pine savannas, pine flatwoods, sandhills, and pine-oak forests within the Coastal Plains of southeastern North America [26] and is considered a specialist of Longleaf Pine (*Pinus palustris*) forests by some [42].

### 2.2. Methodological overview

Following the methods of Crawford et al. [31], we estimated the probability of persistence of the Ornate Chorus Frog across its distribution at locations where the species has previously been observed [43]. We gathered georeferenced records of Ornate Chorus Frog presence from various sources that spanned 187 years (1836–2024; section 2.3). We buffered each observation by 2.5 km and dissolved overlapping buffered observations into 407 populations, which assumed that observations less than 5 km apart were part of the same population (section 2.4). We built detection histories for each population based on the observation records. Most of our data were presence-only, and in most cases, we did not have data on places that were searched but Ornate Chorus Frogs were not detected. Thus, we built search-effort histories for each population based on detections of non-target species within each of the 407 population polygons (section 2.5). Specifically, we assumed that an observer who reported a specific non-target species near a location where Ornate Chorus Frogs had previously been reported would have also reported Ornate Chorus Frogs if they had been seen or heard during the same search event. We used the detection histories of the target species, search effort histories of the non-target species, and environmental covariates (section 2.7) to model the probability of persistence in 2024 of each Ornate Chorus Frog population using the modified Cormack-Jolly-Seber model presented by Crawford et al. [31], which estimates the probability that a population persists in 2024, analogous to survival probability (section 2.6).

### 2.3. Ornate Chorus Frog presence data

We compiled Ornate Chorus Frog observations from across the historical distribution using records from Global Biodiversity Information Facility (GBIF) and museums, Natural Heritage databases [44–46], community science observations (iNaturalist and Herpmapper), the North American Amphibian Monitoring Program [47], previously conducted surveys [27], and the primary literature (S1 Appendix in S1 File). These data came from visual or auditory observations of individuals, dipnet surveys for tadpoles, drift fence surveys, call surveys conducted at road stops, data from acoustic recording units, and opportunistic observations.

We omitted records that fell >10 km outside of the Gap Analysis Project Species Range Map for Ornate Chorus Frogs [48], as these locations were likely erroneous. We also omitted records that had an estimate of location uncertainty >5 km. We note that 36% of the records in the cleaned dataset did not have an estimate of location uncertainty; we retained these records but acknowledge that the unknown spatial error might add noise. We omitted records that were likely duplicates because the same observation was reported in >1 database or when more than one frog was reported from the same observation event and recorded separately. Specifically, we filtered out records with both an identical day of observation and coordinates that were within 10 m of another record; the 10-m cut-off helped to identify some duplicate records with coordinates that had been rounded.

### 2.4. Defining Ornate Chorus Frog populations

We defined population boundaries by adding a 2.5-km radius buffer around each Ornate Chorus Frog observation and then dissolving the perimeters of the overlapping buffers into one polygon [31]. These polygons represent both 1) populations, whereby we assume individuals or observations within the same polygon are not independent and 2) the area for which a detection of a non-target species could reasonably represent search effort for Ornate Chorus Frogs (i.e., we assume that detection of a non-target species within a population polygon is equivalent to a non-detection of Ornate Chorus Frog; section 2.5). We conducted a sensitivity analysis, whereby we varied the buffer size (1-km and 5-km radius buffers) and repeated the analysis to ensure our conclusions were not sensitive to this decision (S2 Appendix in S1 File). For context, Crawford et al. [31] used 5-km buffers to delineate Southern Hognose Snake (*Heterodon simus*) populations. Note that by grouping nearby observations into populations, we are unable to detect metapopulation dynamics or extirpation at individual wetlands within the population boundary. We then assigned each Ornate Chorus Frog population centroid to one of three level III ecoregions [31,49]. Population centroids occurred in the Southern Coastal Plain, Middle Atlantic Coastal Plain, and Southeastern Plains ecoregions.

### 2.5. Estimating search effort history using observations of non-target species

We used records of eight non-target anuran species with distributions that overlap with portions of the Ornate Chorus Frog distribution [28,50] to index search effort for Ornate Chorus Frogs. Following Crawford et al. [31], we assumed that if an observer reported one of the eight non-target species to a database such as HerpMapper or iNaturalist, they would also have reported an Ornate Chorus Frog if they had also observed it. In other words, we used records of the non-target species within Ornate Chorus Frog polygons as an index of search effort. We used the North American Amphibian Monitoring Program database (NAAMP) [47] to guide our selection of non-target species: we pooled anuran observations in the NAAMP database over state and year and calculated the number of times that a particular non-target species was observed or heard calling when the Ornate Chorus Frog was also heard calling. For example, the Spring Peeper (*P. crucifer*) was heard calling at the same time and the same location as 83% of all Ornate Chorus Frog observations. In other words, if Ornate Chorus Frogs were heard, Spring Peepers were also heard 83% of the time (refer to S3 Appendix in S1 File for other species associations). Thus, we selected eight species that we assumed would be active and detectable during the same peak activity time of Ornate Chorus Frogs based on the NAAMP data: Spring Peeper, Southern Chorus Frog (*P. nigrita*), Little Grass Frog (*P. ocularis*), Brimley’s Chorus Frog (*P. brimleyi*; in South Carolina only, based on higher co-occurrences in SC in the NAAMP database (S3 Appendix in S1 File)), Upland Chorus Frog (*P. feriarum*; in South Carolina only), Southern Leopard Frog (*Lithobates sphenocephalus*), Green Frog (*L. clamitans*; in Mississippi only), and Cope’s Gray Treefrog (*Dryophytes chrysoscelis*; in Mississippi only; S3 Appendix in S1 File). We also included all observations of the Ornate Chorus Frog in our search effort history as these detections also represent search events. Finally, we included records of Flatwoods Salamander (*Ambystoma cingulatum/bishopi*) larvae from the Florida Fish and Wildlife Conservation Commission (FWC) dipnet surveys for winter-breeding amphibians in Florida (described in S1 Appendix in S1 File); Enge et al. [27] noted that Ornate Chorus Frog larvae were found in 63% of the ponds where Flatwoods Salamander larvae were also found (12 of 19 ponds), thus we used the presence of Flatwoods Salamanders in the FWC database (*n* = 78 records, 2010–2017) as an additional index of search effort.

We obtained records of non-target species from community science databases (iNaturalist (downloaded on 15 Aug 2024) and HerpMapper), the Global Biodiversity Information Facility [51–59] (downloaded on 20 Aug 2024), the North American Amphibian Monitoring Program [47], and the FWC dipnet surveys for winter-breeding amphibians (Florida only; refer to S1 Appendix in S1 File for details). We filtered the non-target species observation data to include only records observed during the peak months of Ornate Chorus Frog detectability: between Nov 1 and Apr 30, dates when Ornate Chorus Frogs are likely calling or observed moving to or from breeding ponds, or when Ornate Chorus Frog tadpoles should be present. We removed records that represented duplicate effort (i.e., records from the exact same date, coordinates, and observer, regardless of species observed). Further, we filtered observations of non-target species to exclude records with >2.5 km location error or with no estimate of error. Finally, we retained only those records that were observed within each Ornate Chorus Frog population (i.e., within the 2.5-km dissolved buffers). In total, we compiled 21,424 records of the eight non-target species and Ornate Chorus Frog, of which 4,376 records were within Ornate Chorus Frog population polygons. Observation records of non-target species, excluding the Ornate Chorus Frog observations, ranged from 1923–2024, and 87% (of 2,418 records) were observed on or after the year 2000.

### 2.6. Persistence model

We used the persistence model developed by Crawford et al. [31] to estimate the probability that historical Ornate Chorus Frog populations currently persist in 2024; please refer to Crawford et al. [31] for a detailed description of the modeling process. In Crawford et al. [31], the persistence of a population (analogous to survival of an individual) was estimated using the Cormack-Jolly-Seber model [60,61] from detection histories using a Bayesian framework with Markov chain Monte Carlo methods (MCMC) [62]. We modeled the persistence state history of each Ornate Chorus Frog population over the period from 1950 to 2024. Like Crawford et al. [31], we assumed that populations observed prior to 1950 were present in 1950 and that when an Ornate Chorus Frog was first documented in a new location after 1950, that it had been present but undetected in that location in 1950 (i.e., we assumed no colonization of new areas after 1950). The model also assumed that each population had persisted each year between 1950 and the year of last observation. State histories of each population were modeled using a Bernoulli trial with an annual population-specific persistence probability, where a population could either persist or become extirpated given that it persisted the year before, with the observation process modeled by the detection probability of Ornate Chorus Frog.

Following Crawford et al. [31], we modeled detection as a logit linear function with fixed-effect parameters for the linear temporal trend in detection and search effort. The predictors were year and search effort history (the scaled number of observer-days), specific to each population and year. Also following Crawford et al. [31], we modeled persistence as a logit-linear function of three spatial covariates that did not vary by time: the average habitat suitability within a population polygon (section 2.7.1), the average winter precipitation within a population polygon (section 2.7.2), and the proportion of impervious land cover within a population polygon (section 2.7.3). We also included an ecoregion-specific intercept in our model to account for similar ecological characteristics, such as soil and geology, within an ecoregion (section 2.4). Because we derived the spatial covariates from relatively recent datasets, we assumed that these conditions were representative of conditions that have influenced persistence since 1950.

Our modeling process, like Crawford et al. [31], began with uninformative priors for all parameters, and we generated three MCMC chains using 100,000 iterations where we retained every third iteration from the last 50,000 iterations, yielding a final set of 50,001 samples from posterior distributions of the parameters. We visually inspected chain mixing to confirm convergence using MCMC trace plots and assessed whether Brooks-Gelman-Rubin statistics were < 1.1 for all parameters. Like Crawford et al. [31], we also assessed model fit by conducting posterior predictive checks of Ornate Chorus frog detections by comparing the mean number of populations with detections from simulated data to the real data in three distinct time periods (1970–1974, 2000–2004, and 2020–2024). We calculated the posterior means and 95% credible intervals for each parameter, and when the credible intervals did not overlap 0, we interpreted this as an important impact. We conducted our analyses in Program R [63] using the packages R2jags [64], coda [65], lattice [66], and tidyverse [67].

### 2.7. Covariates

We selected three landscape-scale variables as predictors in the persistence model based on what is known about the environmental conditions that support Ornate Chorus Frog populations [26,27,30,38].

#### 2.7.1. Suitable land cover.

We considered suitable habitat to be that which is necessary to support Ornate Chorus Frogs during both the breeding and non-breeding seasons. Thus, we used recent (since 2010) observations of Ornate Chorus Frogs and a maximum entropy (MaxEnt) model [68,69] to create a raster representing relative habitat suitability for Ornate Chorus Frogs across their distribution (described in S4 Appendix in S1 File). The model included five variables: percent sand within the top 25-cm horizon, the proportion of coniferous, mixed, and shrub-scrub forest, and the proportion of land classified as small ponds or wetlands (<20 ha in area; S4 Appendix in S1 File). Recognizing that most Ornate Chorus Frogs are observed during the breeding season, we transformed each land cover variable to best capture suitable land cover for Ornate Chorus Frogs for all life stages. Specifically, we created a new raster for each variable such that each pixel in the raster represented the average value of all pixels within a 450 m neighborhood (S4 Appendix in S1 File). We used 450 m to encompass both the location error that could be present around each observation (recall that we retained observations with ≤ 100 m location error) and the upper limits of the distance that Ornate Chorus Frogs travel between breeding and non-breeding locations (~ 450 m observed max; [27,30]).

**Fig 1:**
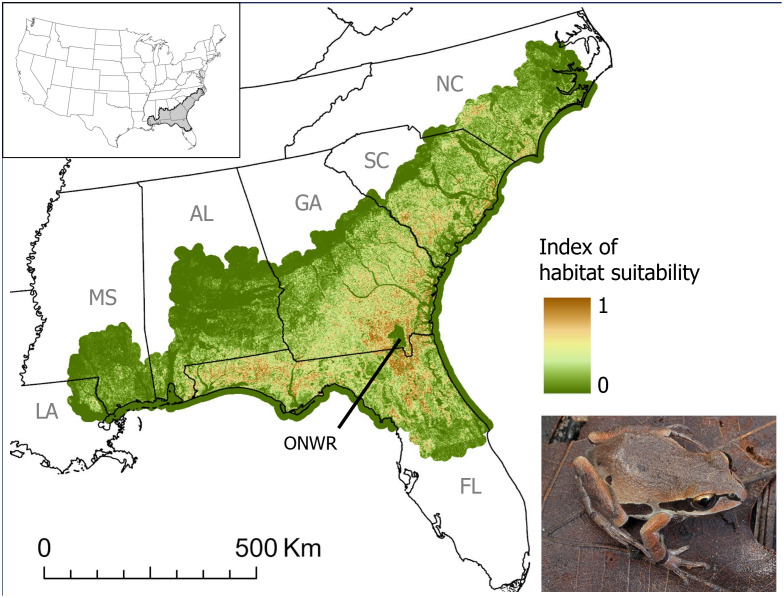
Ornate Chorus Frog habitat suitability. Relative habitat suitability for Ornate Chorus Frogs (*Pseudacris ornata*) in the southeastern USA, based on MaxEnt, where brown represents relatively high suitability and green represents relatively low suitability (refer to S4 Appendix in S1 File). The outer polygon represents the Gap Analysis Project Species Range Map for Ornate Chorus Frogs (CC0 1.0) [48] with a 10-km-wide buffer. The location of Okefenokee National Wildlife Refuge (ONWR) is noted. Photo credit to Alan Cressler (public domain). North America basemap (CC BY 4.0) is from the Commission for Environmental Cooperation [70].

Our model performed well: the area under the receiver operating characteristic curve (AUC) was 0.87 (S4 Appendix in S1 File). Sandy soil and coniferous forest were the most important variables (S4 Appendix in S1 File). We used the final model to compute a complimentary log-log (cloglog) transformation as a 30m by 30m continuous raster to represent the predicted probability of suitable conditions for the species across the distribution (i.e., habitat suitability), with values ranging from 0–1 (Fig 1). From this raster, we calculated the average habitat suitability within each Ornate Chorus Frog population polygon by finding the average pixel value within the 2.5-km dissolved buffers.

#### 2.7.2. Mean winter precipitation.

We downloaded gridded (5 km) monthly precipitation data from the National Oceanic and Atmospheric Administration (NOAA) Monthly U.S. Climate Gridded Dataset (NClimGrid) [71]. For each grid cell, we calculated the mean winter precipitation (Dec–Feb inclusive) per cell from the last 30 years (1993–2023). We scaled the data so that they ranged between 0 and 1 and then calculated the mean winter precipitation within each Ornate Chorus Frog population polygon.

#### 2.7.3. Urban impervious surfaces.

We calculated average imperviousness with the 2021 National Land Cover Database (NLCD) urban impervious surface layer [72]. Each grid cell in this 30 m raster dataset represents the percent of that cell that is impervious. We calculated the average value of the impervious surface pixels, ranging from 0–1, within each Ornate Chorus Frog population polygon.

#### 2.7.4. Correlations.

After calculating average covariate values within Ornate Chorus Frog populations, we used the corrr package (v 0.4.4) [73] to calculate Pearson correlation coefficients among continuous covariates. There was no notable correlation between average habitat suitability and average imperviousness (*r* = −0.29) or between average winter precipitation and either average habitat suitability (*r* = −0.27) or average imperviousness (*r* = −0.13).

### 2.8. Population persistence in protected areas

We used the Protected Areas Database of the United States (PAD-US 4.0) [74] to delineate protected areas within our study area. We grouped protected areas in the database that intersected with the centroids of the 407 Ornate Chorus Frog populations into eight categories: state forests (*n* = 15 Ornate Chorus Frog populations), state parks (*n* = 3 populations), national forests (*n* = 37 populations), national wildlife refuges (*n* = 10 populations), wildlife management areas (*n* = 14 populations), lands dedicated to U.S. military installations (*n* = 24 populations), non-government organization and privately owned land (e.g., The Nature Conservancy, Ducks Unlimited, Tall Timbers Research Station & Land Conservancy; *n* = 21) and ‘other’ (*n* = 37 populations; included land owned by, for example, regional water management districts, U.S. Department of Energy (specifically Savannah River Site, SC), and Estuarine Research Reserves). Lands dedicated to U.S. military installations are managed to optimize both the conservation of natural resources and the mission requirements of the Department of Defense and other land use activities [75]. We then calculated the average probability of persistence in 2024 across Ornate Chorus Frog populations located within protected areas. We compared the mean probability of persistence of populations within protected areas (pooled over the eight categories) to populations located outside of protected areas (*n* = 267).

## 3. Results

**Fig 2:**
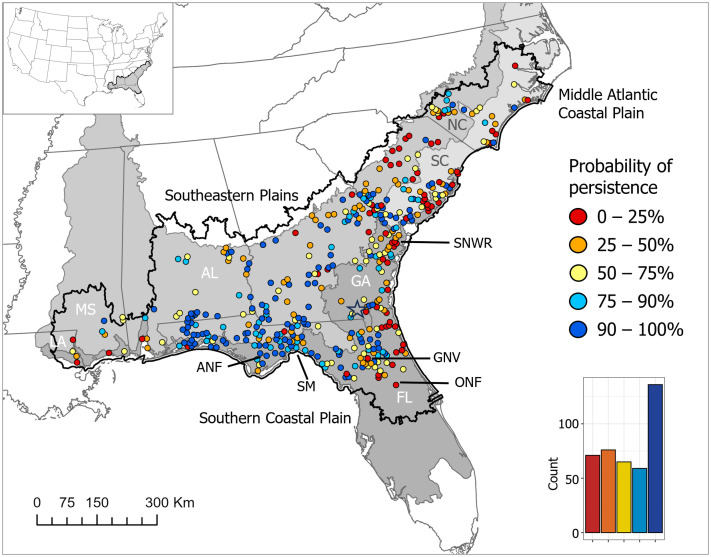
Probability of persistence. Probability of persistence of Ornate Chorus Frog (*Pseudacris ornata*) populations (n = 407) across the distribution (heavy black line; (CC0 1.0) [48]) in the southeastern USA. Shading indicates Level III ecoregions (public domain) [49]. Locations noted in the text include the Apalachicola National Forest (ANF), St. Marks National Wildlife Refuge (SM), the city of Gainesville (GNV), Ocala National Forest (ONF), Savannah National Wildlife Refuge (SNWR), and Okefenokee National Wildlife Refuge (hollow star). The histogram shows the number of populations in each probability of persistence category, with categories corresponding to the colors in the map legend. North America basemap (CC BY 4.0) is from the Commission for Environmental Cooperation [70].

Our cleaned dataset of Ornate Chorus Frog observations included 2227 records spanning seven states (FL, GA, SC, NC, AL, MS, LA) and 187 years (1836–2024; Table 1). We grouped records into 407 population polygons that ranged in area from 19.9 to 385.7 km^2^ (mean 19.9 km^2^, SD = 32.0 km^2^). Population polygons with the largest areas occurred where there were many observations (S2 Appendix in S1 File); for example, the largest polygon was located in the Apalachicola National Forest in the panhandle of Florida and the second-largest polygon was located near the city of Gainesville, FL (note that many of the records in the latter polygon were from the 1930s, with the last record in 1972). Populations occurred in three level III ecoregions: the Southern Coastal Plain (*n* = 161), the Southeastern Plains (*n* = 192), and the Middle Atlantic Coastal Plain (*n* = 54; Fig 2).

**Table 1:** Number of Ornate Chorus Frog (*Pseudacris ornata*) records (total *n* = 2227) and populations^a^ per state in our cleaned dataset, and the probability that populations still persist in 2024.

State	Number of records	Date range of records	Number of populations	Probability of persistence		Proportion of populations with mean probability < 0.5
				Mean	SD	
Florida	1202	1901–2024	144	0.72	0.30	0.25
Alabama	89	1905–2024	34	0.71	0.26	0.24
Georgia	478	1929–2024	98	0.62	0.30	0.39
Mississippi	8	1934–2007	7	0.59	0.27	0.29
North Carolina	100	1933–2023	32	0.53	0.28	0.50
South Carolina	338	1836–2024	88	0.52	0.35	0.50
Louisiana	12	1926–1954	4	0.37	0.24	0.75

^a^We defined populations by adding a 2.5-km radius buffer to each record in the cleaned dataset and dissolving overlapping buffers; the resulting polygons each represented one of 407 population.

There was a total of 4,376 records of non-target species and Ornate Chorus Frog that fell within the 407 population polygons, with a mean of 10.7 records per polygon (SD = 31.6, min = 1, max = 455). Thirty-nine percent (160 of 407) of the population polygons had only one observation (in those cases, a single observation of Ornate Chorus Frog and no observations of non-target species; S2 Appendix in S1 File). The estimated mean detection rate of Ornate Chorus Frog in a population that was estimated to still persist was 0.014 (Table 2). We found a positive linear temporal trend and a positive effect of search effort on Ornate Chorus Frog detection rate (Table 2, S5 Appendix in S1 File). Probability of persistence was positively correlated with the date of the most recent Ornate Chorus Frog observation (Pearson *r* = 0.88; S5 Appendix in S1 File).

**Table 2:** Parameter estimates (means and 95% Bayesian credible intervals (2.5 to 97.5 percentile of the posterior distribution)) for the model predicting the probability that 407 historical Ornate Chorus Frog (*Pseudacris ornata*) populations still persist between 1950 and 2024. We interpret mean parameter estimates with credible intervals that do not overlap zero as ecologically important.

Parameter^a^		Mean	Lower 95%	Upper 95%
Persistence				
*μ*_*ecoregion*_	Southeastern Plains	0.808	0.530	0.958
	Southern Coastal Plain	0.815	0.587	0.947
	Middle Atlantic Coastal Plain	0.834	0.600	0.961
*β*_*HS*_		2.856	1.311	4.648
*β*_*rain*_		4.007	2.093	6.007
*β*_*impervious*_		−1.553	−2.509	−0.550
Detection				
*μ*_*mean*_		0.014	0.012	0.017
*β*_*trend*_		0.019	0.015	0.023
*β*_*effort*_		0.279	0.236	0.322

^a^*β* – covariate effects on persistence and detection; *μ* – intercept; HS – habitat suitability; rain – average winter rainfall; impervious – percent of the surface that is impervious.

The mean probability of persistence in 2024 across all 407 Ornate Chorus Frog populations was 0.63 (SD = 0.32; Fig 2). More than 36% (147 of 407) of historical Ornate Chorus Frog populations had a probability of persistence in 2024 of < 0.50, and 17% (71 of 407) had a probability of persistence of < 0.25. Conversely, more than 33% (136 of 407) of the historical Ornate Chorus Frog populations had a probability of persistence in 2024 of > 0.90. Mean annual persistence rates were similar across ecoregions (ecoregion-specific intercepts ranged from 0.81–0.83; Table 2). The mean probability of persistence in 2024 across populations within an ecoregion, however, was higher in the Southeastern Plains ecoregion (mean = 0.69, SD = 0.30, *n* = 192) than in either the Southern Coastal Plain ecoregion (mean = 0.58, SD = 0.33, *n* = 161; *p* = 0.02) or the Middle Atlantic Coastal Plain ecoregion (mean = 0.59, SD = 0.31, *n* = 54; p = 0.05; Fig 2). In the Southern Coastal Plain ecoregion, 23.6% (38 of 161) of the populations had a probability of persistence < 0.25; many of these populations were located in peninsular Florida and along the Atlantic coast in Georgia and South Carolina (Fig 2).

**Fig 3:**
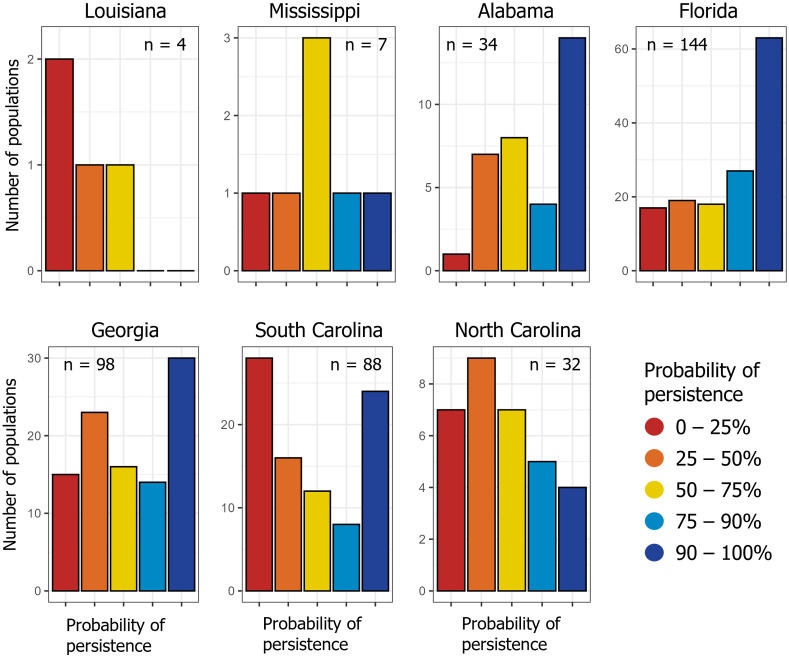
Number of Ornate Chorus Frog (*Pseudacris ornata*) populations per state within each category of persistence probability. For example, in Florida we identified 144 Ornate Chorus Frog populations, of which 63 populations had a > 90% probability of still persisting in 2024 and 17 had < 25% probability of still persisting in 2024. Sample size is the number of historical populations that we have identified in each state. Note that the y-axis limits differ in each panel. Refer to Fig 2 for a spatial representation of these data.

We compared the mean probability of persistence in 2024 across populations within each state. Mean probability of persistence was relatively low for the four Ornate Chorus Frog populations in Louisiana (mean = 0.37, SD = 0.24, *n* = 4 populations, Table 1, Fig 3). The mean probability of persistence in South Carolina (mean = 0.52, SD = 0.35, *n* = 88 populations) and North Carolina (mean = 0.53, SD = 0.28, *n* = 32) was also relatively low (Table 1, Fig 3). In both South Carolina and North Carolina, 50% of the Ornate Chorus Frog populations (44 of 88 in SC and 16 of 32 in NC) had a probability of persistence ≤ 0.50 (Figs 2 and 3). The mean (SD) probability of persistence in Mississippi was 0.59 (0.27); only two of the seven populations in Mississippi had a probability of persistence > 0.75, with last known observations in Mississippi, to our knowledge, in 2006 and 2007. Georgia had the second highest number of observations in the cleaned dataset (478 observations in 98 populations) and a mean (SD) probability of persistence of 0.62 (0.30; Table 1, Fig 3). Almost 40% of historical Ornate Chorus Frog populations in Georgia had a probability of persistence of < 0.5; many of these were along Georgia’s Atlantic coast. Florida and Alabama had the highest mean (SD) probability of persistence, 0.72 (0.30) and 0.71 (0.26), respectively; Table 1, Fig 3). Northwestern Florida (otherwise known as the panhandle region of Florida), south-central Alabama, southwestern Georgia, and southwestern South Carolina appear to be the species’ strongholds (Fig 2).

Probability of population persistence was positively influenced by mean winter precipitation and habitat suitability (Table 2, S6 Appendix in S1 File), with sandy soil and coniferous forest being the most important variables contributing to our model of suitable habitat (S4 Appendix in S1 File). The probability of persistence was negatively influenced by the percent of the surface that was impervious (Table 2, S6 Appendix in S1 File). Historical populations that now have the highest mean percent imperviousness include population polygons in Jacksonville FL (46.0% impervious), Columbia SC (41.3% impervious), Mobile AL (40.2% impervious), and Savannah GA (32.5% impervious); there was a relatively low probability of Ornate Chorus Frog persistence (< 0.03; S6 Appendix in S1 File) in these four regions.

### 3.1. Population persistence in protected areas

**Fig 4:**
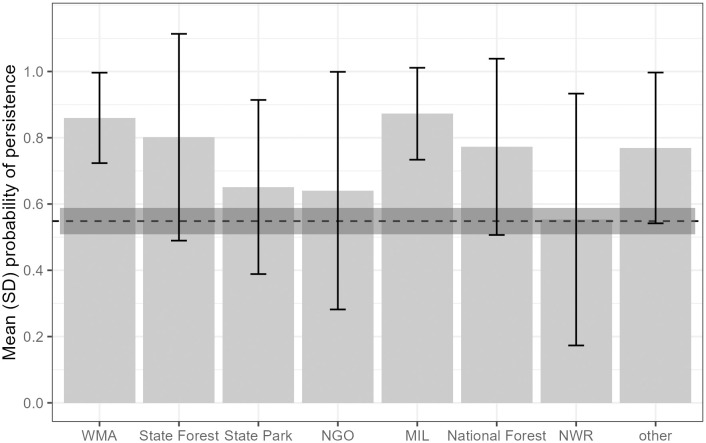
Mean (SD) probability of persistence across Ornate Chorus Frog (*Pseudacris ornata*) populations throughout the distribution in the southeastern USA by type of protected area. For context, the horizonal line represents the mean probability of persistence of populations located on non-protected land (mean = 0.55, *n* = 246 Ornate Chorus Frog populations), with the 95% confidence interval in dark grey. The x-axis shows wildlife management areas (WMA; *n* = 14 Ornate Chorus Frog populations), state forests (*n* = 15), state parks (*n* = 3), non-government organizations and privately owned land (NGO; *n* = 21), lands dedicated to U.S. military installations (MIL; *n* = 24), national forests (*n* = 37 populations), national wildlife refuges (NWR; *n* = 10 populations), and ‘other’ (*n* = 37; section 2.8).

We found that, overall, the mean probability of persistence was significantly higher for populations in protected areas (mean = 0.76, SD = 0.27, *n* = 161) compared to populations that occurred on lands that were not protected (mean = 0.55, SD = 0.32, *n* = 246; *t* = 7.33, df = 378, *p* < 0.001). National and state forests, wildlife management areas, and U.S. military installations had relatively high probability of persistence (> 0.77; Fig 4). Mean probability of persistence was relatively high on Federally-owned protected lands in general, except for populations in national wildlife refuges, which had a relatively low average probability of persistence but high variation (mean = 0.55, SD = 0.38, min = 0.01, max = 0.99, *n* = 10; Fig 4). Across these refuges, populations in St. Marks National Wildlife Refuge, FL, had a relatively high probability of persistence (mean = 0.93, SD = 0.06, *n* = 4), whereas populations in Okefenokee National Wildlife Refuge, GA, had a relatively low probability (mean = 0.47, SD = 0.23, *n* = 3; Figs 2 and 4). Ornate Chorus Frog populations in and around the Savannah National Wildlife Refuge that spans the border of northeastern Georgia and southeastern South Carolina have not been detected, to our knowledge, since the 1960s (Fig 2). Mean habitat suitability was higher in population polygons that were within protected areas compared to those outside of protected areas (t = −4.29, df = 338, p < 0.001), and mean percent impervious was lower for population polygons within protected areas compared to those outside of protected areas (*t* = 6.61, df = 293, *p* < 0.001; S6 Appendix in S1 File). Mean winter precipitation within population polygons did not differ between protected and non-protected areas (*t* = −1.21, df = 340, *p* = 0.23; S6 Appendix in S1 File).

### 3.2. Sensitivity

Our overall conclusions were not sensitive to the size of the buffer around Ornate Chorus Frog observations (S2 Appendix in S1 File). When we used 1-km buffers or 5-km buffers, we also found that the probability of persistence in 2024 was positively influenced by habitat suitability and mean winter precipitation and negatively influenced by the imperviousness of the surface (S2 Appendix in S1 File). Regardless of the buffer size, historical populations of Ornate Chorus Frogs that occurred in what are now the cities of Jacksonville FL, Gainesville FL, Mobile AL, and Savannah GA, for example, have the lowest probability of persistence in 2024. Similarly, regardless of buffer size, populations of Ornate Chorus Frogs in, for example, Fort Stewart GA, Apalachicola National Forest FL, and in properties owned by the Jones Center at Ichauway and The Orianne Society, both in Georgia, had among the highest probabilities of persistence.

## 4. Discussion

We found that range wide, more than 36% of the historical Ornate Chorus frog populations that we identified are possibly extirpated (probability of persistence < 0.50) and more than 17% are likely extirpated (probability of persistence < 0.25). Many of these populations are along the Atlantic coasts of Florida, Georgia, South Carolina, and North Carolina, as well as central South Carolina and Louisiana. Conversely, more than 47% of Ornate Chorus Frog populations likely still persist (probability > 0.75) and more than 33% have a probability of persistence of > 0.90. Many of the extant populations are clustered in northwestern Florida (the panhandle), southwestern Georgia, and southeastern Alabama. Populations were more likely to persist in areas with higher habitat suitability and higher winter rainfall and less likely to persist in areas where urban imperviousness was relatively high. Ornate Chorus Frog populations located within protected areas, such as national forests and wildlife management areas, had a higher average probability of persistence compared to populations on non-protected land.

Ornate Chorus Frog populations that are likely extirpated occurred in every state within the species’ distribution. In Louisiana, the presence of Ornate Chorus frogs has not been verified since the 1950s, and the species is thought to be extirpated in the state [28,29]. Ornate Chorus Frogs are rare in Mississippi, with no verified records to our knowledge since one Ornate Chorus Frog was heard calling at a military training facility in Mississippi in 2007 [76]. The two extant populations that our study identified in Mississippi likely represent the westernmost edge of the distribution and could be isolated by ~100 km from neighboring populations in Alabama. Our habitat suitability map predicted that the amount of suitable habitat is relatively low in these westernmost portions of the range. Ornate Chorus Frogs are likely extirpated through much of their historical range in peninsular Florida; populations near Ocala National Forest, FL, for example, have not been detected since the 1970s. In North Carolina, where the Ornate Chorus Frog is state-listed as an endangered species, our records search uncovered relatively few observations, of which half are possibly or likely extirpated.

Despite likely extirpation of historical Ornate Chorus Frog populations across their distribution, there are many locations where Ornate Chorus Frog populations are still present. Ornate Chorus Frogs were observed in northern portions of peninsular Florida (e.g., [77]), throughout much of the distribution in Georgia, and in South Carolina near its border with Georgia. In southcentral Alabama, southwestern Georgia, and throughout much of Florida’s panhandle region, Ornate Chorus Frog populations have a high probability of persisting, and indeed, this region appears to be the species’ stronghold [27]. Our habitat suitability map suggested highly suitable land cover in this region, consisting of sandy soils and coniferous forest; here, clusters of pine savanna ecosystems are relatively abundant [78]. We acknowledge that the relatively large number of Ornate Chorus Frog observations in Florida could reflect more intensive search effort and not necessarily higher abundance in Florida. Our dataset of Ornate Chorus Frog observations included the extensive work by Enge et al. [27] to gather unpublished survey data in Florida. The outcome of this higher search effort is that we likely have a more accurate picture of the species’ status in Florida relative to other states. Uneven search effort across space in general could result in a lower probability of detection in areas that are not searched well, and extant populations could be misidentified as unlikely to persist (i.e., false negative). Thus, more search effort in parts of the range that have not been searched recently could improve the accuracy of persistence estimates.

We found that current site conditions (habitat suitability, winter rainfall, and imperviousness of the surface) impact the probability that a population persists in 2024, and conversely, the probability that a population is extinct. This generally concurs with what is known about Ornate Chorus Frogs and their association with pine savannas and the sandy soils needed for burrowing during the non-breeding season. Abundant winter rain ensures that ephemeral breeding ponds have sufficient water to sustain tadpoles until metamorphosis. Land-use changes from suitable to impervious surfaces is associated with the extinction of Ornate Chorus Frog populations that occurred there historically. There are areas within the distribution that have relatively high habitat suitability but low probability of persistence; these tend to occur in the outskirts of cities, especially near the cities of Jacksonville and Gainesville, FL. Especially in the Gainesville area, observations of non-target species are abundant, suggesting that neither search effort nor land-use change, at least at the scale that we have measured it, have played a role in population extirpation in that area. There are also areas of relatively high habitat suitability within the distribution that have no recent Ornate Chorus Frog observations to our knowledge. For example, areas surrounding the Okefenokee National Wildlife Refuge in Georgia have few recent Ornate Chorus Frog observations, despite relatively high predicted habitat suitability. This region also has abundant floodplains and wetlands dominated by Pond Cypress (*Taxodium ascendens*) and Bald Cypress (*T. distichum*) that tend to be poorly drained, suggesting that our habitat suitability model may be overestimating the suitability of habitat for Ornate Chorus Frogs in this region. Relatively few roads in this area could mean lower search effort and thus a lower probability of detection, although one population polygon on the east side of the refuge had several non-target species observations.

We found that Ornate Chorus Frog populations that were within protected areas, such as national and state forests, wildlife management areas, and U.S. military installations, tended to have a higher probability of persistence than populations in non-protected areas. In North Carolina, four of the nine likely extant populations (probability > 0.75) were located on or adjacent to land owned by military operations. Protected areas likely have fewer threats to Ornate Chorus Frog populations from land use change that can impact both breeding and non-breeding habitat, as well as habitat connectivity that can support recolonization following local extirpation. In some parts of the distribution, however, we found that some Ornate Chorus Frog populations have a low probability of persistence despite occurring within protected areas, such as populations in Ocala National Forest in peninsular Florida and Croatan National Forest in NC. Mean probability of persistence in national wildlife refuges was lower than areas outside of protected areas, driven by low probabilities of extant Ornate Chorus Frog populations in some refuges, such as Okefenokee National Wildlife Refuge in GA, the Savannah National Wildlife Refuge in both GA and SC, and Santee National Wildlife Refuge in SC, where predicted habitat suitability for the species is low.

The modeling approach that we used capitalized on occurrence records of target and non-target species from museum and community science databases to identify regions across the distribution where Ornate Chorus Frogs occurred at one time but are now likely extirpated. This information can be used for evaluating range-wide extinction risk for rare and at-risk species, especially species for which range-wide survey data are lacking [31]. Our study results are aligned with the current NatureServe [29] ranks in each state and may prompt consideration by individual states to assess species status and enact state-level protections, similar to North Carolina’s ‘endangered’ designation for Ornate Chorus Frogs in the state. This methodological approach, which allows for incorporation of species presence data from a variety of sources, could be particularly useful for assessing the range-wide status of species that are otherwise data-deficient.

There are several limitations of the modeling approach that we used, in addition to the possibility of misidentifying extant populations as extinct because the area has not been searched recently (note that we detected a positive effect of search effort on the probability of detection). First, we used land cover data that reflect recent conditions, limiting our ability to model the effect of landscape conditions at the time that a population may have become extirpated. Second, we assumed that all populations had been present in 1950 regardless of when the population was first discovered. Thus, we were unable to detect colonization, range expansion, or metapopulation dynamics that could be occurring. Third, we found that detection probability increased over time; similar to Crawford et al. [31], it is possible that the temporal trend and search effort parameters were correlated. Indeed, the number of species observations increased over time in our dataset. Finally, search effort was low for many population polygons, with 39% of polygons having only one observation. By increasing buffer size in our sensitivity analysis, we effectively increased search effort per polygon and we found that our overall conclusions were not sensitive to this change. We note, however, that although polygons with recent Ornate Chorus Frog observations tended to have a higher persistence probability, some polygons with only one observation had a higher probability of persistence than one might expect given the date at which Ornate Chorus Frogs were last observed there. For example, one population polygon with low search effort in Louisiana had a probability of persistence of 0.68, despite the last known observation occurring in 1936. Whether low search effort can result in inflated persistence probabilities could be an avenue of future investigation.

There are other threats to Ornate Chorus Frog population persistence that we did not include in our models but that could have an unmeasured impact on persistence. For example, we did not assess the role of disease in driving low probability of persistence or presumed extirpation across the range of the Ornate Chorus Frog. Chytridiomycosis and ranavirus disease have been associated with mass mortality events of amphibians [10,17,79]. Both pathogens have been detected in Ornate Chorus Frogs, but neither have been known to cause mass die-offs in the species [80]. Multi-year drought also has potential to impact population persistence of Ornate Chorus Frogs, and we did not have data to model this variable at a population level. Ornate Chorus Frogs require specific wetland conditions to support breeding, and prolonged drought that impacts the hydroperiod of ephemeral ponds can delay breeding or result in reproductive failure [26,27,38,41,81]. This, coupled with a nearly annual population turnover, where adults typically reproduce once during their only year of life [38], means that multiple consecutive years of drought and associated reproductive failure can result in population extirpation, especially if there are few nearby refuges to act as sources for recolonization [82]. We were also unable to model the impacts of invasive or predatory species on Ornate Chorus Frog population persistence at a range-wide scale. Davis et al. [40] showed a negative association between Ornate Chorus Frog presence and predatory fish in breeding ponds, and further, that flooding caused by extreme weather events is a mechanism by which predatory fish can be introduced into wetlands. Finally, we were unable to model the impacts of local habitat alterations, such as wetland loss or fire suppression, that alter the integrity of pine savanna landscapes and wetland hydroperiod, impacting reproductive success and suitability of breeding and non-breeding habitat [83]. Fire suppression allows woody vegetation to encroach on herbaceous wetlands, negatively impacting microhabitat suitability [84,85]. Future research could investigate the impacts of these variables on the probability of Ornate Chorus Frog population persistence. Further, additional research could project extinction risk of Ornate Chorus Frog populations into the future given predictions of future changes in winter rainfall patterns, land-use change, and continued urbanization (e.g., [31,86,87]).

Our study highlights the magnitude at which population extirpation has likely occurred across the Ornate Chorus Frog distribution since 1950. This information can be used by wildlife managers, as well as stewards of public and private land, to identify regions with at-risk populations, and restore and conserve a network of heterogeneous wetlands to support breeding and recruitment, as well as upland habitats suitable for burrowing during the non-breeding season. In parts of the distribution where Ornate Chorus Frogs populations appear stable, management approaches may include conserving the integrity of breeding and non-breeding habitat. Our study also identifies areas with suitable Ornate Chorus Frog habitat that may have extant populations that are yet unconfirmed; these regions could be prioritized for surveys. Finally, our study emphasizes the role of protected areas and private lands in maintaining population persistence. The Ornate Chorus Frog is ranked among the most intrinsically sensitive anuran species in North America [25], yet little is known about its range-wide population status. Our study fills a knowledge gap by identifying regions where Ornate Chorus Frog populations are likely thriving and regions where they may be extinct.

## Supporting information

S1 File S1 Appendix. Sources of Ornate Chorus Frog observation records. S2 Appendix. Examples of three buffer sizes to delineate Ornate Chorus Frog populations and sensitivity of persistence models to buffer size. S3 Appendix. Using the North American Amphibian Monitoring Program database to guide selection of non-target species to be used as an index of search effort. S4 Appendix. Predicting environmental suitability for Ornate Chorus Frogs using MaxEnt. S5 Appendix. Number of species detections per year (1900–2024) and relationship between observation date and persistence probability. S6 Appendix. Impact of predictor variables on probability of persistence.
(ZIP)
