# Supplementary material for: Persistence of a declining anuran species across its distribution
Source: PLoS One. 2025 Sep 22;20(9):e0332991. doi: 10.1371/journal.pone.0332991 (PMC12453189; doi:10.1371/journal.pone.0332991)
Supplement: S1 File — S1 Appendix. Sources of Ornate Chorus Frog observation records. S2 Appendix. Examples of three buffer sizes to delineate Ornate Chorus Frog populations and sensitivity of persistence models to buffer size. S3 Appendix. Using the North American Amphibian Monitoring Program database to guide selection of non-target species to be used as an index of search effort. S4 Appendix. Predicting environmental suitability for Ornate Chorus Frogs using MaxEnt. S5 Appendix. Number of species detections per year (1900–2024) and relationship between observation date and persistence probability. S6 Appendix. Impact of predictor variables on probability of persistence. (ZIP) [file pone.0332991.s001.zip › supporting_information_R1_clean/S1_Appendix.docx]

Supplementary information supporting:

Persistence of a declining anuran species across its distribution

Erin L. Koen^1^, E. Hance Ellington^2,3^, William J. Barichivich^4^, Howard Kochman^4^, Kevin M. Enge^5^, and Susan C. Walls^4^

^1^ Cherokee Nation System Solutions, contracted to, U.S. Geological Survey, Wetland and Aquatic Research Center, Gainesville, Florida, USA, ^2^ Range Cattle Research and Education Center, University of Florida, Ona, Florida, USA, ^3^ Department of Wildlife Ecology and Conservation, University of Florida, Gainesville, Florida, USA, ^4^ U.S. Geological Survey, Wetland and Aquatic Research Center, Gainesville, Florida, USA, ^5^ Fish and Wildlife Research Institute, Florida Fish and Wildlife Conservation Commission, Gainesville, Florida, United States of America

# S1 Appendix. Sources of Ornate Chorus Frog observation records

We compiled records of Ornate Chorus Frog (*Pseudacris ornata*) observations from eight sources:

*1. Global Biodiversity Information Facility (GBIF).* The GBIF is a global-scale data infrastructure for biodiversity data. These data are compiled from numerous sources, including natural history museums, community science databases, literature, and DNA sequencing. We downloaded records of *P. ornata* on 23 Jan 2023 (GBIF.org 2024). 26% of the 2,365 records included the date of observation and a detailed description of the observation location but did not include coordinates. Thus, we used the location description to estimate the coordinates of 627 records (166 of which were unique locations) using Geolocate (Rios and Bart 2018) or other mapping software. For the observations that we georeferenced, we conservatively estimated location uncertainty in meters as a radius around the coordinates (Wieczorek et al. 2004).

*2. Museum records*. We obtained records of *P. ornata* in Feb 2023 directly from two museums because coordinates for museum records were not available in the GBIF dataset: the American Museum of Natural History (n = 107; 9 unique locations) and the North Carolina Museum of Natural Sciences (n = 599; 149 unique locations). We retained only those records that contained both the year of observation and location coordinates or a detailed description of the location. For records without coordinates but with a detailed description of the location, we estimated the coordinates using Geolocate (Rios and Bart 2018) or other mapping software, and we estimated location uncertainty in meters.

*3. Natural Heritage Program databases*. We acquired state-wide records of *P. ornata* from Natural Heritage Program databases in South Carolina (n = 26 unique locations; South Carolina Heritage Trust Program 2023), Alabama (n = 26 unique locations; State of Alabama, Department of Conservation and Natural Resources 2023), and Louisiana (n = 1 unique location; Louisiana Department of Wildlife and Fisheries Wildlife Diversity Program 2023). The Ornate Chorus Frog is not tracked by Natural Heritage Programs in Georgia and Florida. Sample sizes noted above represent records that were unique to these datasets and not present in GBIF or other publicly available databases.

*4. Community science observations*. We compiled opportunistic observations of *P. ornata* reported by the public to the online databases iNaturalist (www.inaturalist.org) and HerpMapper (www.herpmapper.org). For iNaturalist observations, we retained only those records considered research grade (i.e., identification has been verified by the naturalist community). The majority (87%) of the research-grade records of *P. ornata* in the iNaturalist database had coordinates that were obscured (30-km error) to protect the location of this rare species. Thus, we retained only those records for which the true coordinates were available (n = 24 unique observations) or for which we were able to obtain the true coordinates by communicating directly with the observers (n = 92 additional unique locations). We considered records reported to iNaturualist on or before 23 August 2024 and we requested georeferenced *P. ornata* records from HerpMapper on 10 March 2023.

*5. North American Amphibian Monitoring Program (NAAMP)*. The NAAMP was a U.S. Geological Survey (USGS) program run in collaboration with state agencies, universities, and non-government organizations (U.S. Geological Survey 2017). For 21 years (1994–2015), call surveys for breeding amphibians were conducted at stops along routes across North America (Refer to USGS (2017) for protocol details). We compiled 187 records of *P. ornata* reported in the NAAMP database (2002–2015).

*6. VertNet*. VertNet is a repository of biodiversity data containing records from scientific collections worldwide (VertNet 2016). We downloaded all records of *P. ornata* on 19 Jan 2023 and retained all records that were not also present in other databases (n = 2).

*7. Dipnet survey data*. The Florida Fish and Wildlife Conservation Commission FWC conducted dipnet surveys for winter-breeding amphibians between 2010 and 2014 at 931 unique ponds in Florida (refer to Enge et al. 2014 for details); we retained 1,607 unique *P. ornata* records from this database (2006–2022). The FWC also compiled unpublished survey and museum record data for winter-breeding amphibians in Florida, as well as observations from Owen (1996), into a database, described in Enge et al. (2014). This second database included additional records of *P. ornata* in Florida from dipnet surveys for tadpoles, drift fence surveys, call surveys conducted at road stops, data from acoustic recording units, and opportunistic observations; we also included these records in our database (n = 534 *P. ornata* records from 1922 to 2022). Note that we removed duplicate records from the amalgamated dataset by identifying records obtained on the same date and within 10 m of one another (refer to section 2.3 of the main text).

The Jones Center at Ichauway, a 29,000 acre property in southwestern Georgia, shared amphibian dipnet survey data (2002–2020). We retained 142 Ornate Chorus Frog records from this data set.

*8. Observations reported in published literature*. We searched published papers that contained details of the date and location of *P. ornata* observations by searching both Google Scholar and specifically the journal Herpetological Review on 19 January 2023 using the term “*Pseudacris ornata”*. These searches resulted in 181 journal articles, 55 of which contained relevant data (i.e., date of observation and coordinates or description of location). We repeated these searches on 15 August 2024 using the same search term and retrieved one additional record. From the 56 articles with relevant data (S1Table), we extracted the date and county of 112 unique *P. ornata* observations described in the literature, and we estimated the coordinates using Geolocate software (Rios and Bart 2018) or Google Maps (maps.google.com) based on location descriptions when exact coordinates were not provided in the text. We estimated location uncertainty in meters for the observations that we georeferenced. Any use of trade, firm, or product names is for descriptive purposes only and does not imply endorsement by the U.S. Government.

S1 Table. Records of *Pseudacris ornata* observations that we gathered from published literature^a^.

| 1 | Alford, R. A. (1986). Habitat use and positional behavior of anuran larvae in a northern Florida temporary pond. Copeia, 408–423. |
| --- | --- |
| 2 | Alix, D. M., Anderson, C. J., Grand, J. B., & Guyer, C. (2014). Evaluating the effects of land use on headwater wetland amphibian assemblages in coastal Alabama. Wetlands, 34, 917–926. |
| 3 | Blouin, M. S. (1989). Life history correlates of a color polymorphism in the ornate chorus frog, *Pseudacris ornata*. Copeia, 319–325. |
| 4 | Brandt, B. B. (1936). The frogs and toads of eastern North Carolina. Copeia, 4, 215–223. |
| 5 | Brandt, B. B. (1953). Salientia of Bleckley County, Georgia, and vicinity. Herpetologica, 9(3), 141–145. |
| 6 | Brown, L. E., & Means, D. B. (1984). Fossorial behavior and ecology of the chorus frog *Pseudacris ornata*. Amphibia-Reptilia, 5(3–4), 261–273. |
| 7 | Burrow, A. K., & Maerz, J. C. (2021). Experimental confirmation of effects of leaf litter type and light on tadpole performance for two priority amphibians. Ecosphere, 12(9), e03729. |
| 8 | Burrow, A. K., Crawford, B. A., & Maerz, J. C. (2021). Ground cover and native ant predation influence survival of metamorphic amphibians in a Southeastern pine savanna undergoing restoration. Restoration Ecology, 29(7), e13410. |
| 9 | Carr, A. F. (1940). Dates of frog choruses in Florida. Copeia, 1940(1), 55–55. |
| 10 | Chandler, H. C. (2015). The effects of climate change and long-term fire suppression on ephemeral pond communities in the southeastern United States (Doctoral dissertation, Virginia Tech). |
| 11 | Davis, J. C., Castleberry, S. B., and Kilgo, J.C. (2010). Influence of coarse woody debris on herpetofaunal communities in upland pine stands of the southeastern Coastal Plain. Forest Ecology and Management, 1111–1117. |
| 12 | Davis, C. L., Miller, D. A., Walls, S. C., Barichivich, W. J., Riley, J. W., & Brown, M. E. (2017). Species interactions and the effects of climate variability on a wetland amphibian metacommunity. Ecological Applications, 27(1), 285–296. |
| 13 | Degner, J. F., Silva, D. M., Hether, T. D., Daza, J. M., & Hoffman, E. A. (2010). Fat frogs, mobile genes: unexpected phylogeographic patterns for the ornate chorus frog (*Pseudacris ornata*). Molecular Ecology, 19(12), 2501–2515. |
| 14 | DeGregorio, B. A., Willson, J. D., Dorcas, M. E., & Gibbons, J. W. (2014). Commercial value of amphibians produced from an isolated wetland. The American Midland Naturalist, 172(1), 200–204. |
| 15 | Dye, M. L., Fedler, M. T., & Enge, K. M. (2022). *Pseudacris ornata* – Ornate Chorus Frog. Herpetological Review 53(4), 627. |
| 16 | Eason, G. W., & Fauth, J. E. (2001). Ecological correlates of anuran species richness in temporary pools: a field study in South Carolina, USA. Israel Journal of Zoology, 47(4), 346–365. |
| 17 | Enge, K. M., & Marion, W. R. (1986). Effects of clearcutting and site preparation on herpetofauna of a north Florida flatwoods. Forest Ecology and Management, 14(3), 177–192. |
| 18 | Erwin, K. J., Chandler, H. C., Palis, J. G., Gorman, T. A., & Haas, C. A. (2016). Herpetofaunal communities in ephemeral wetlands embedded within longleaf pine flatwoods of the Gulf Coastal Plain. Southeastern Naturalist, 15(3), 431–447. |
| 19 | Farmer, A. L. (2014). Geographic Distribution: *Pseudacris ornata* (ornate chorus frog). Herpetological Review 45(3): 459. |
| 20 | Farmer, A., Smith, L., Gibbons, J. W., & Castleberry, S. (2009). A comparison of techniques for sampling amphibians in isolated wetlands in Georgia, USA. Applied Herpetology, 6(4), 327–341. |
| 21 | Funderburg, J. B. (1955). The amphibians of New Hanover County, North Carolina. Journal of the Elisha Mitchell Scientific Society, 71(1), 19–28. |
| 22 | Goff, C. B., Walls, S. C., Rodriguez, D., & Gabor, C. R. (2020). Changes in physiology and microbial diversity in larval ornate chorus frogs are associated with habitat quality. Conservation Physiology, 8(1), coaa047. |
| 23 | Goldberg, C. S., Strickler, K. M., & Fremier, A. K. (2018). Degradation and dispersion limit environmental DNA detection of rare amphibians in wetlands: Increasing efficacy of sampling designs. Science of the Total Environment, 633, 695–703. |
| 24 | Graham, S. P., & Gray, K. M. (2009). Geographic Distribution: *Pseudacris ornata* (ornate chorus frog). Herpetological Review 40(4), 447. |
| 25 | Hanlin, H. G., Martin, F. D., Wike, L. D., & Bennett, S. H. (2000). Terrestrial activity, abundance and species richness of amphibians in managed forests in South Carolina. The American Midland Naturalist, 143(1), 70–83. |
| 26 | Harkey, G. A., & Semlitsch, R. D. (1988). Effects of temperature on growth, development, and color polymorphism in the ornate chorus frog *Pseudacris ornata*. Copeia, 1001–1007. |
| 27 | Harper, F. (1931). Notes on two Georgia species of *Pseudacris*. Copeia, 4, 159–161. |
| 28 | Harper, F. (1937). A season with Holbrook's chorus frog (*Pseudacris ornata*). American Midland Naturalist, 18(2), 260–272. |
| 29 | Hill, R. L., & Levy, M. G. (2014). Prevalence of *Batrachochytrium dendrobatidis* in pond-breeding amphibians of the fall line Sandhills region of Georgia, USA. Herpetological Review, 45(2), 238–240. |
| 30 | Holt, B. (2017). Geographic Distribution: *Pseudacris ornata* (ornate chorus frog). Herpetological Review, 48(1), 142 |
| 31 | Holt, B. (2017). Geographic Distribution: *Pseudacris ornata* (ornate chorus frog). Herpetological Review, 48(3), 586 |
| 32 | Jobson, H. G. (1940). Reptiles and amphibians from Georgetown County, South Carolina. Herpetologica, 39–43. |
| 33 | Knapp, D. D., Smith, L. L., & Atkinson, C. L. (2021). Larval anurans follow predictions of stoichiometric theory: Implications for nutrient storage in wetlands. Ecosphere, 12(4), e03466. |
| 34 | Ledvina, J. A. (2008). Responses of upland herpetofauna to the restoration of Carolina bays and thinning of forested bay margins (Doctoral dissertation, Clemson University). |
| 35 | Lee, J. R. (2009). The herpetofauna of the Camp Shelby Joint Forces Training Center in the Gulf Coastal Plain of Mississippi. Southeastern Naturalist, 8(4), 639–652. |
| 36 | Lemmon, E. M., Lemmon, A. R., Collins, J. T., Lee-Yaw, J. A., & Cannatella, D. C. (2007). Phylogeny-based delimitation of species boundaries and contact zones in the trilling chorus frogs (*Pseudacris*). Molecular Phylogenetics and Evolution, 44(3), 1068–1082. |
| 37 | Love, C. N., Winzeler, M. E., Beasley, R., Scott, D. E., Nunziata, S. O., & Lance, S. L. (2016). Patterns of amphibian infection prevalence across wetlands on the Savannah River Site, South Carolina, USA. Diseases of Aquatic Organisms, 121(1), 1–14. |
| 38 | McLendon, J. P., Hanlin, H. G., & Nelson, E. A. (1996). Reptile and amphibian characterization of a thermally disturbed braided stream undergoing restoration near Aiken, SC. In Proceedings of the 23rd Annual Conference on Ecosystem Restoration and Creation (pp. 228–41). |
| 39 | Mecham, J. S. (1965). Genetic relationships and reproductive isolation in southeastern frogs of the genera *Pseudacris* and *Hyla*. American Midland Naturalist, 269–308. |
| 40 | Moseley, K. R., Castleberry, S. B., & Schweitzer, S. H. (2003). Effects of prescribed fire on herpetofauna in bottomland hardwood forests. Southeastern Naturalist, 2(4), 475–486. |
| 41 | Neill, W. T. (1957). Notes on metamorphic and breeding aggregations of the eastern spadefoot, *Scaphiopus holbrooki* (Harlan). Herpetologica, *13*(3), 185–187. |
| 42 | Pechmann, J. H., Scott, D. E., Gibbons, J. W., & Semlitsch, R. D. (1989). Influence of wetland hydroperiod on diversity and abundance of metamorphosing juvenile amphibians. Wetlands Ecology and Management, 1(1), 3–11. |
| 43 | Pechmann, J. H., Scott, D. E., Semlitsch, R. D., Caldwell, J. P., Vitt, L. J., & Gibbons, J. W. (1991). Declining amphibian populations: the problem of separating human impacts from natural fluctuations. Science, 253(5022), 892–895. |
| 44 | Quinby, J. A. (1954). Interesting breeding dates for some South Carolina frogs. Herpetologica, 10(1), 8–8. |
| 45 | Schwartz, A., & Etheridge, R. (1954). New and additional herpetological records from the North Carolina Coastal Plain. Herpetologica, 10(3), 167–171. |
| 46 | Sekerak, C. M., Tanner, G. W., & Palis, J. G. (1996). Ecology of flatwoods salamander larvae in breeding ponds in Apalachicola National Forest. In Proceedings of the Annual Conference of the Southeastern Association of Fish and Wildlife Agencies, 50, 321–330. |
| 47 | Semlitsch, R. D., Scott, D. E., Pechmann, J. H. K., & Gibbons, J. W. (1996). Structure and dynamics of an amphibian community. Long-term studies of vertebrate communities, 217–248. |
| 48 | Snodgrass, J. W., Komoroski, M. J., Bryan Jr, A. L., & Burger, J. (2000). Relationships among isolated wetland size, hydroperiod, and amphibian species richness: implications for wetland regulations. Conservation Biology, 14(2), 414–419. |
| 49 | Steen, D. A., Rall McGee, A. E., Hermann, S. M., Stiles, J. A., Stiles, S. H., & Guyer, C. (2010). Effects of forest management on amphibians and reptiles: generalist species obscure trends among native forest associates. Open Environmental Sciences, 4(1). |
| 50 | Stevenson, D. (2011). Geographic distribution: *Pseudacris ornata* (ornate chorus frog). Herpetological Review, 42(2), 252. |
| 51 | Stevenson, D., & Jenkins, C. (2011). Geographic distribution: *Pseudacris ornata* (ornate chorus frog). Herpetological Review, 42(2), 252. |
| 52 | Taylor, W., Stohlgren, K. & Stevenson, D. (2011). Geographic distribution: *Pseudacris ornata* (ornate chorus frog). Herpetological Review, 42(2), 256. |
| 53 | Todd, B. D., & Winne, C. T. (2006). Ontogenetic and interspecific variation in timing of movement and responses to climatic factors during migrations by pond-breeding amphibians. Canadian Journal of Zoology, 84(5), 715–722. |
| 54 | Todd, B. D., Luhring, T. M., Rothermel, B. B., & Gibbons, J. W. (2009). Effects of forest removal on amphibian migrations: implications for habitat and landscape connectivity. Journal of Applied Ecology, 554–561. |
| 55 | Travis, J., & Trexler, J. C. (1984). Investigations on the control of the color polymorphism in *Pseudacris ornata*. Herpetologica, 252–257. |
| 56 | Viosca, P. (1938). Notes on the winter frogs of Alabama. Copeia, 4, 201–201. |

^a^We searched Google Scholar and the journal Herpetological Review on 15 August 2024 using the term “*Pseudacris ornata”*. This table includes only those articles with a unique observation date and a location description that we could estimate coordinates from. Several of these records were also present in museum records; we identified and removed duplicates from the amalgamated dataset.

**References**

Enge, K. M., Farmer, A. L., Mays, J. D., Castellon, T. D., Hill, E. P., & Moler, P. E. (2014). Survey of winter-breeding amphibian species. Final report. Florida Fish and Wildlife Conservation Commission. Fish and Wildlife Research Institute, Wildlife Research Section, Gainesville, 136.

GBIF.org (2024a). GBIF Occurrence Download 20 Aug 2024 – *Pseudacris ornata*. https://doi.org/10.15468/dl.m24bac.

Louisiana Department of Wildlife and Fisheries Wildlife Diversity Program. (2023). Biotics 5 database [Dev. NatureServe, Arlington, Virginia]. LDWF, Baton Rouge, Louisiana. (Accessed: June 20, 2023).

Owen, R. D. (1996). Breeding phenology and microhabitat use among three chorus frog species (Pseudacris) in east-central Florida. M.S. Thesis, University Central Florida, Orlando, Florida, USA. 256pp.

Rios, N. E., & Bart Jr, H. L. (2018). GEOlocate: a platform for georeferencing natural history collections data. Available at https://geo-locate.org/default.html

South Carolina Heritage Trust Program. (2023). South Carolina Department of Natural Resources. Accessed May 2023.

State of Alabama, Department of Conservation and Natural Resources. (2023). Heritage Data Collection. Available at https://heritage.dcnr.alabama.gov/. Accessed: 26 May 2023.

U.S. Geological Survey. (2017). North American Amphibian Monitoring Program (NAAMP) anuran detection data from the eastern and central United States (1994–2015). Available at [https://www.usgs.gov/data/north-american-amphibian-monitoring-program-naamp-anuran-detection-data-eastern-and-central. 10.5066/F7G44NG0](https://www.usgs.gov/data/north-american-amphibian-monitoring-program-naamp-anuran-detection-data-eastern-and-central.%2010.5066/F7G44NG0)

VertNet. (2016). VertNet home page. Available from: vertnet.org. [22 May 2023].

Wieczorek, J., Guo, Q., & Hijmans, R. (2004). The point-radius method for georeferencing locality descriptions and calculating associated uncertainty. International Journal of Geographical Information Science, 18(8), 745–767.
