# Supplementary material for: Persistence of a declining anuran species across its distribution
Source: PLoS One. 2025 Sep 22;20(9):e0332991. doi: 10.1371/journal.pone.0332991 (PMC12453189; doi:10.1371/journal.pone.0332991)
Supplement: S1 File — S1 Appendix. Sources of Ornate Chorus Frog observation records. S2 Appendix. Examples of three buffer sizes to delineate Ornate Chorus Frog populations and sensitivity of persistence models to buffer size. S3 Appendix. Using the North American Amphibian Monitoring Program database to guide selection of non-target species to be used as an index of search effort. S4 Appendix. Predicting environmental suitability for Ornate Chorus Frogs using MaxEnt. S5 Appendix. Number of species detections per year (1900–2024) and relationship between observation date and persistence probability. S6 Appendix. Impact of predictor variables on probability of persistence. (ZIP) [file pone.0332991.s001.zip › supporting_information_R1_clean/S2_Appendix.docx]

Supplementary information supporting:

Persistence of a declining anuran species across its distribution

Erin L. Koen^1^, E. Hance Ellington^2,3^, William J. Barichivich^4^, Howard Kochman^4^, Kevin M. Enge^5^, and Susan C. Walls^4^

^1^ Cherokee Nation System Solutions, contracted to, U.S. Geological Survey, Wetland and Aquatic Research Center, Gainesville, Florida, USA, ^2^ Range Cattle Research and Education Center, University of Florida, Ona, Florida, USA, ^3^ Department of Wildlife Ecology and Conservation, University of Florida, Gainesville, Florida, USA, ^4^ U.S. Geological Survey, Wetland and Aquatic Research Center, Gainesville, Florida, USA, ^5^ Fish and Wildlife Research Institute, Florida Fish and Wildlife Conservation Commission, Gainesville, Florida, United States of America

# S2 Appendix. Examples of three buffer sizes to delineate Ornate Chorus Frog populations and sensitivity of persistence models to buffer size.


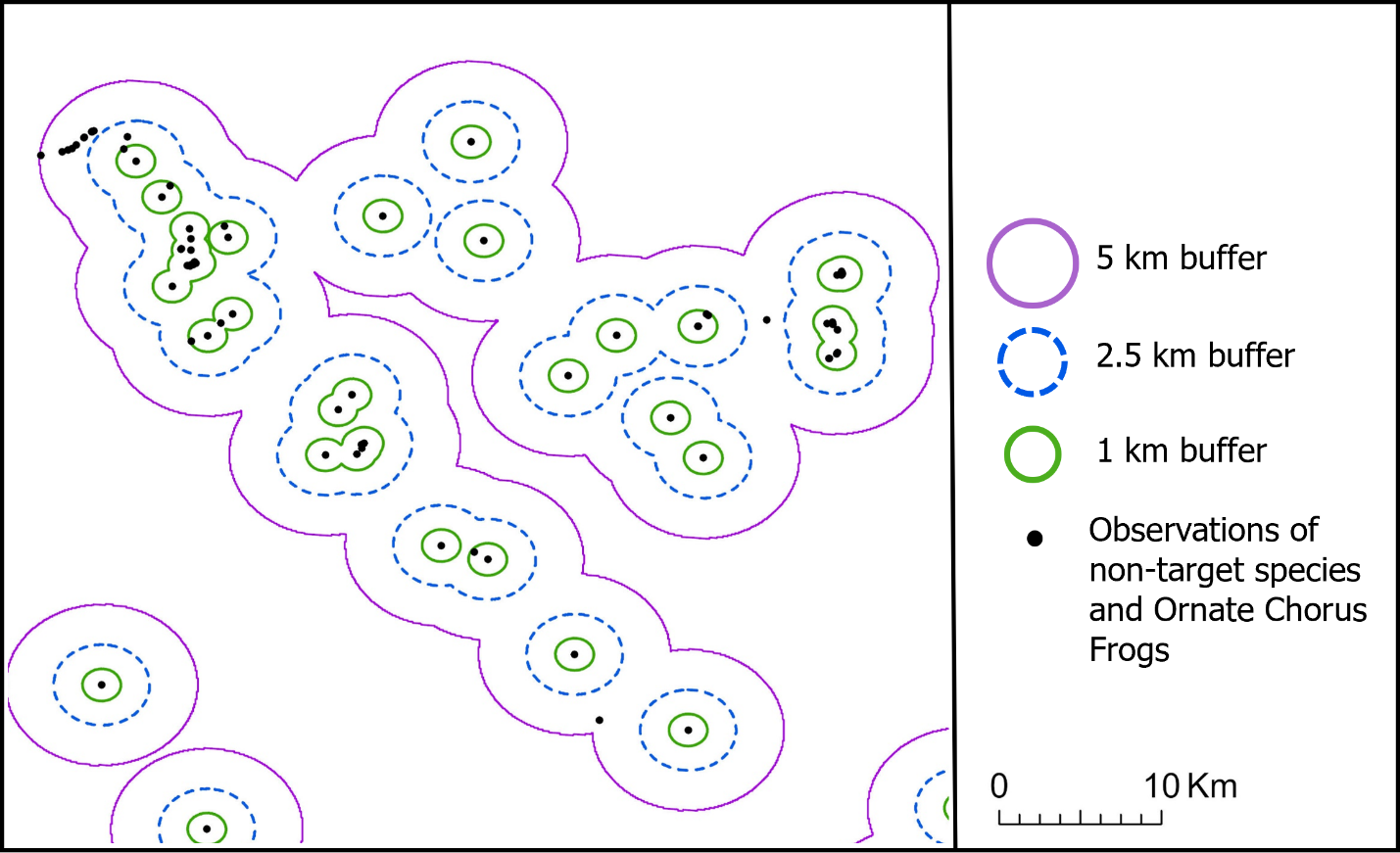


S1 Figure. Examples of the three buffer sizes around Ornate Chorus Frog (*Pseudacris ornata*) observations. The upper left population polygon is considered one population when using a 5-km radius buffer, 11 populations when using a 2.5-km radius buffer, and 21 populations when using a 1-km radius buffer. We used the 2.5-km buffer for our models, but we showed in S2 Table that the trends are similar regardless of buffer size.

S2 Table. A sensitivity analysis for different buffer sizes around each Ornate Chorus Frog observation record. Values in the 2.5-km buffer column are the same as reported in the main text; we repeated them here to ease comparison with the other buffer sizes.

|  | | 1 km buffer | 2.5 km buffer | 5 km buffer |
| --- | --- | --- | --- | --- |
| Number of populations | | 638 | 407 | 243 |
| Number of populations with one record only | | 413 (65%) | 160 (39%) | 76 (31%) |
| Number of populations per ecoregion | Southeastern Plains | 291 | 192 | 124 |
|  | Southern Coastal Plain | 249 | 161 | 87 |
|  | Middle Atlantic Coastal Plain | 98 | 54 | 32 |
| Area of population polygons (km^2^) | Mean | 4.7 | 33.6 | 167.0 |
|  | SD | 7.6 | 32.0 | 195.6 |
|  | Min. | 3.2 | 19.9 | 79.5 |
|  | Max. | 167.5 | 385.7 | 1251.3 |
| Total number of non-target species^a^ records that fell within a population polygon | | 3713 | 4378 | 5246 |
| Non-target records per population | Mean | 5.8 | 10.7 | 21.4 |
|  | SD | 20.2 | 31.6 | 51.7 |
|  | Min. | 1 | 1 | 1 |
|  | Max. | 426 | 455 | 513 |
| Probability of persistence | Mean | 0.78 | 0.63 | 0.46 |
|  | SD | 0.16 | 0.14 | 0.14 |
|  | Min. | 0.000 | 0.003 | 0.001 |
|  | Max. | 1.0 | 1.0 | 1.0 |
| Persistence (95% credible interval in parentheses)^b^ | | | | |
| *β_HS_* | | 3.74 (2.18–5.51) | 2.86 (1.31–4.65) | 2.22 (0.90–3.58) |
| *β_rain_* | | 4.40 (2.35–6.36) | 4.01 (2.09–6.01) | 2.95 (1.21–4.68) |
| *β_impervious_* | | -2.77 (-4.02– -1.47) | -1.55 (-2.51– -0.55) | -1.36 (-2.49– -0.16) |
| Detection (95% credible interval in parentheses) | | | | |
| *μ_mean_* | | 0.010 (0.009–0.012) | 0.014 (0.012–0.017) | 0.022 (0.019–0.027) |
| *β_trend_* | | 0.018 (0.015–0.021) | 0.019 (0.015–0.023) | 0.019 (0.015–0.024) |
| *β_effort_* | | 0.507 (0.449–0.568) | 0.279 (0.236–0.322) | 0.223 (0.192–0.255) |

^a^the number of non-target species records includes those of eight non-target species as well as Ornate Chorus Frog records.

^b^*β* – covariate effects on persistence and detection; *μ* – intercept. HS – habitat suitability; rain – average winter rainfall; impervious – percent of the surface that is impervious.
