# Supplementary material for: Persistence of a declining anuran species across its distribution
Source: PLoS One. 2025 Sep 22;20(9):e0332991. doi: 10.1371/journal.pone.0332991 (PMC12453189; doi:10.1371/journal.pone.0332991)
Supplement: S1 File — S1 Appendix. Sources of Ornate Chorus Frog observation records. S2 Appendix. Examples of three buffer sizes to delineate Ornate Chorus Frog populations and sensitivity of persistence models to buffer size. S3 Appendix. Using the North American Amphibian Monitoring Program database to guide selection of non-target species to be used as an index of search effort. S4 Appendix. Predicting environmental suitability for Ornate Chorus Frogs using MaxEnt. S5 Appendix. Number of species detections per year (1900–2024) and relationship between observation date and persistence probability. S6 Appendix. Impact of predictor variables on probability of persistence. (ZIP) [file pone.0332991.s001.zip › supporting_information_R1_clean/S3_Appendix.docx]

Supplementary information supporting:

Persistence of a declining anuran species across its distribution

Erin L. Koen^1^, E. Hance Ellington^2,3^, William J. Barichivich^4^, Howard Kochman^4^, Kevin M. Enge^5^, and Susan C. Walls^4^

^1^ Cherokee Nation System Solutions, contracted to, U.S. Geological Survey, Wetland and Aquatic Research Center, Gainesville, Florida, USA, ^2^ Range Cattle Research and Education Center, University of Florida, Ona, Florida, USA, ^3^ Department of Wildlife Ecology and Conservation, University of Florida, Gainesville, Florida, USA, ^4^ U.S. Geological Survey, Wetland and Aquatic Research Center, Gainesville, Florida, USA, ^5^ Fish and Wildlife Research Institute, Florida Fish and Wildlife Conservation Commission, Gainesville, Florida, United States of America

# S3 Appendix. Using the North American Amphibian Monitoring Program database to guide selection of non-target species to be used as an index of search effort

S3 Table. The percentage of Ornate Chorus Frog (*Pseudacris ornata*) observations (*n* = 187) for which other anuran species were also heard calling^1^. Data are summarized from the North American Amphibian Monitoring Program (NAAMP; USGS 2017). We present proportions pooled across years (2002–2015) and across the distribution^2^ of *P. ornata*, as well as proportions by state^3^ (number in brackets is the total number of *P. ornata* detections in the database per state, pooled across years).

| Non-target species | Common name | Percent of *P. ornata* observations that a non-target species was also heard calling^1^ | | | | | |
| --- | --- | --- | --- | --- | --- | --- | --- |
|  |  | Pooled over distribution | NC (6) | SC  (100) | GA  (50) | FL (25) | MS (6) |
| *Pseudacris crucifer* | Spring peeper | 83.4 | 66.7 | 95.0 | 80.0 | 64.0 | 16.7 |
| *P. ocularis* | Little grass frog | 9.1 | 0 | 10.0 | 4.0 | 20.0 | NA |
| *P. nigrita* | Southern chorus frog | 51.3 | 0 | 66.0 | 44.0 | 32.0 | 0 |
| *P. brimleyi*^4^ | Brimley’s chorus frog | 9.1 | 0 | 17.0 | 0 | NA | NA |
| *P. feriarum*^4^ | Upland chorus frog | 17.1 | 0 | 26.0 | 12.0 | 0 | 0 |
| *Lithobates sphenocephalus* | Southern leopard frog | 49.7 | 16.7 | 55.0 | 46.0 | 52.0 | 16.7 |
| *L. clamitans*^5^ | Green frog | 1.6 | 0 | 0 | 0 | 4.0 | 33.3 |
| *Dryophytes chrysoscelis*^5^ | Cope’s gray treefrog | 5.3 | 0 | 5.0 | 4.0 | 0 | 50.0 |

^1^ For example, if *P. ornata* was heard calling at a road stop, *P. crucifer* was also heard 83% of the time.

^2^ North Carolina (NC), South Carolina (SC), Georgia (GA), Florida (FL), and Mississippi (MS). ^3^ There were zero *P. ornata* detections in Louisiana or in Alabama in the NAAMP surveys. We used ‘NA’ to indicate that a particular non-target species does not occur in that state.

^4^ We considered *P. brimleyi* and *P. feriarum* as non-target species in South Carolina only (even though they occur in other states).

^5^ We considered *L. clamitans* and *D. chrysoscelis* as non-target species in Mississippi only (even though they occur in other states).
