# Supplementary material for: Persistence of a declining anuran species across its distribution
Source: PLoS One. 2025 Sep 22;20(9):e0332991. doi: 10.1371/journal.pone.0332991 (PMC12453189; doi:10.1371/journal.pone.0332991)
Supplement: S1 File — S1 Appendix. Sources of Ornate Chorus Frog observation records. S2 Appendix. Examples of three buffer sizes to delineate Ornate Chorus Frog populations and sensitivity of persistence models to buffer size. S3 Appendix. Using the North American Amphibian Monitoring Program database to guide selection of non-target species to be used as an index of search effort. S4 Appendix. Predicting environmental suitability for Ornate Chorus Frogs using MaxEnt. S5 Appendix. Number of species detections per year (1900–2024) and relationship between observation date and persistence probability. S6 Appendix. Impact of predictor variables on probability of persistence. (ZIP) [file pone.0332991.s001.zip › supporting_information_R1_clean/S4_Appendix.docx]

Supplementary information supporting:

Persistence of a declining anuran species across its distribution

Erin L. Koen^1^, E. Hance Ellington^2,3^, William J. Barichivich^4^, Howard Kochman^4^, Kevin M. Enge^5^, and Susan C. Walls^4^

^1^ Cherokee Nation System Solutions, contracted to, U.S. Geological Survey, Wetland and Aquatic Research Center, Gainesville, Florida, USA, ^2^ Range Cattle Research and Education Center, University of Florida, Ona, Florida, USA, ^3^ Department of Wildlife Ecology and Conservation, University of Florida, Gainesville, Florida, USA, ^4^ U.S. Geological Survey, Wetland and Aquatic Research Center, Gainesville, Florida, USA, ^5^ Fish and Wildlife Research Institute, Florida Fish and Wildlife Conservation Commission, Gainesville, Florida, United States of America

# S4 Appendix. Predicting environmental suitability for Ornate Chorus Frogs using MaxEnt

Our overall goal was to produce a raster representing relative habitat suitability for Ornate Chorus Frogs across their distribution. We used this raster as our index of the presence of suitable habitat for Ornate Chorus Frogs in the main paper (section 2.7.1).

**Background.** The Ornate Chorus Frog (*Pseudacris ornata*) is typically associated with sandy, upland soils of the Coastal Plains region of the southeastern USA (Burrow 2022). During the non-breeding season (~ Apr–Oct), this fossorial species spends much of its time burrowed under sandy soil in upland forests such as pine savannas, pine flatwoods, pine plantations, sandhills, and pine-oak forests (Brown and Means 1984, Enge et al. 2014, Burrow 2022). As breeding season approaches, adults emerge and move to nearby breeding sites that can be up to 425 m away (Brown and Means 1984, Enge et al. 2014). Adults choose fishless, temporary pools, preferring ponds that had been dry the previous summer, to lay eggs (Caldwell 1987). Enge et al (2014) found that breeding ponds in Florida ranged from 0.01 to 14 ha (average 0.78, median 0.39). Eggs typically hatch in ~ 7 days, and metamorphosis is complete after ~90 days (Ethier et al. 2021), at which time metamorphs move to upland forests until the next breeding season.

**Modeling overview**. We used recent observations of Ornate Chorus Frogs across their distribution in the southeastern USA and a maximum entropy (MaxEnt) model (Phillips et al. 2006, 2017) to describe environmental suitability for the species. The algorithm uses presence-only data and a set of environmental grid data to produce a raster grid representing the predicted probability that conditions are suitable for the species (Phillips et al. 2006, 2017). We followed the general workflow outlined in Zurell et al. (2020).

In general, detection probability of Ornate Chorus Frogs should be relatively high during the breeding season (Nov–May), when adults and juveniles move to and from breeding sites, when males can be heard calling, and when tadpoles can be detected in breeding ponds. Conversely, detection probability is relatively low during the non-breeding period when individuals are burrowed (Brown and Means 1984). To create predictor variables that account for the low detection probability while individuals are burrowed, for each variable, raster values represented the average conditions within a 450-m radius. Recall that breeding ponds and overwintering sites are thought to be within 425 m of one another (Brown and Means 1984, Enge et al. 2014).

**Observations**. We compiled Ornate Chorus Frog observations from across the species’ distribution from visual or auditory observations of individuals, dipnet surveys for tadpoles, drift fence surveys, call surveys conducted at road stops, data from acoustic recording units, and opportunistic observations, as described in section 2.3 and Appendix 1. For the purposes of the MaxEnt model, we filtered this dataset to include only recent observations (2010‒2024) with < 100 m estimated location error. For observations in the same location (e.g., repeat surveys), we retained the most recent record. We also omitted duplicate records; this included records present in more than one database, as well as duplicates within a database (e.g., when multiple tadpoles were collected from the same pond on the same day and vouchered at a museum). This cleaned dataset had a total of 865 Ornate Chorus Frog observations (Table S4). We then used the spThin package (Aiello-Lammens et al. 2015) in R (version 4.4.0; R Core Team 2024) to further filter the data to exclude records that fell within 120 m of another record, a value greater than the maximum location error around observations and greater than the 30 m pixel size of the environmental variables. This resulted in a total of 403 records in the thinned dataset (Table S4). To assess the sensitivity of our model results to spatial thinning, we repeated the maxent model with an additional, thinned dataset such that observations were > 500 m from another record, which resulted in a total of 314 records. We have assumed that sampling is adequate and representative, and any biases are accounted for or corrected (Zurell et al. 2020).

**Predictor variables**. We selected a suite of five candidate environmental variables that, based on the literature, could represent the land cover necessary to support Ornate Chorus Frogs (described below). We standardized the cell size and spatial extent of the input spatial data by resampling to a common 30-m grid in WGS 1984 (EPSG 4326). We clipped each layer to the Gap Analysis Project Species Range Map for *P. ornata* that was buffered by 10 km (USGS Gap Analysis Project 2018). We turned each variable into a separate, binary raster (see details below), and used focal statistics in ArcGIS Pro (3.0.3) to calculate, for each pixel, the average value within a 450-m neighborhood. We used 450 m to encompass both the location error that could be present around each observation (recall that we retained observations with ≤100 m location error) and the upper limits of the distance that Ornate Chorus Frogs travel between breeding and wintering habitats (~ 425 m observed max). In this way, we attempted to capture environmental conditions for all life stages. All variables were continuous and ranged from 0 to 1, and no variables were correlated (all pairwise correlation coefficients were < 0.4; Table S5). We have assumed that we included all relevant ecological drivers (or proxies) of the species distribution and that our transformation of binary predictors (i.e., average value within a 450-m neighborhood) describes both breeding and non-breeding season habitat (Zurell et al. 2020). Note that we did not include ecoregion in our model, although we did include ecoregion as a factor in the persistence model.

***Forest***. We used the 2021 National Land Cover Database (NLCD; Dewitz 2023) with a 30-m resolution to represent suitable land cover for Ornate Chorus Frogs. From this, we created three vegetation predictors representing the relative amount of: 1) coniferous forest; 2) mixed forest; and 3) shrub-scrub forest. From binary rasters of each land cover type (0,1), we used focal statistics in ArcGIS Pro (3.0.3) to create a new raster whereby each pixel was the average value within a 450-m neighborhood.

***Soil***. We used the gridded Soil Survey Geographic (gSSURGO; Soil Survey Staff 2023) database with a 30-m resolution to index sandy soil conditions used by Ornate Chorus Frogs. We used a raster dataset representing percent sand within the top 25-cm horizon, defined as the weight percentage of mineral particles 0.05 mm to 2.00 mm in equivalent diameter. We then used focal statistics to create a new raster whereby each pixel was the average value within the 450-m neighborhood.

***Wetland***. We used the National Hydrography Dataset (NHD; USGS 2023) to index potential breeding habitat. From the NHD shapefile, we retained ponds and wetlands that were < 20 ha (Enge et al. (2014) found that breeding ponds in Florida ranged from 0.01 to 14 ha). We then converted the shapefile to a binary raster (0, 1) with the same pixel size (30 m) and extent as the other variables and calculated the average value within the 450-m neighborhood of each pixel.

**Model fitting**. We used the dismo package (1.3-14; Hijmans et al. 2023) in R (4.4.1; R Core Team 2024) to implement the Maxent model (maxent.jar; Phillips et al. [internet]). With the exception of the regularization multiplier (or beta multiplier), we used default settings, including auto features that allow the software to tune features (linear, product, quadratic, and hinge) based on model performance (Phillips et al. 2006, Merow et al. 2013). The regularization coefficient is a parameter that helps reduce model complexity and prevent overfitting; values < 1 will produce estimates with a closer fit to the presence records used to fit the model (Merow et al. 2013) and is set by default for each feature class (Phillips and Dudik 2008). The regularization multiplier is a user-specified constant that can be used to tune the regularization coefficient. To identify the most appropriate regularization multiplier for our dataset, we used the ENMeval (v2.0.4; Kass et al. 2021) package in R to test regularization multiplier values between 0.5 and 5 in increments of 0.5. We selected the value with the lowest Akaike Information Criterion score corrected for sample size (AICc) among the models compared; for our dataset, this value was 3. We then tuned the regularization coefficients in our MaxEnt model (run in the dismo package) by this value. We used 10,000 background locations selected randomly from within the distribution of Ornate Chorus Frogs (USGS Gap Analysis Project 2018). Finally, we used the final model to compute a complimentary log-log (cloglog) transformation (with the dismo package) as a 30 m x 30 m continuous grid, to represent the predicted probability that conditions are suitable for the species across the distribution with values ranging from 0 to 1. This index of habitat suitability for the Ornate Chorus Frog (based on the observation dataset thinned to 120 m) is the product we used in section 2.7.1.

**Evaluation**. We evaluated our model for its predictive accuracy using 5-fold cross validation. We split the thinned data into five subsets, and for each of five runs, 80% of the data (n = 322) was used to train the model and 20% (n = 81) was used to test the model. We used the area under the receiver operating characteristic curve (AUC) to evaluate the model’s predictive accuracy. This approach estimates how well the model correctly distinguishes presence from background locations. Values of AUC close to 0.5 represent models that are not different from random, whereas values closer to 1 signify models with a higher chance of ranking a presence point as higher than a background point. We used percent contribution to assess the relative contribution of each environmental variable to model performance.

**Results.** Our model using the thinned (120 m) observation dataset built with linear, product, quadratic, and hinge feature types and a regularization multiplier of 3 yielded an AUC of 0.873. Sandy soil and coniferous forest were the most important variables (Table S6, Figs S2 and S3).

When we used the dataset thinned to 500 m, our results were similar: the AUC was 0.862 and the covariates contributed similarly to the model (Table S6). In Fig S4, we show the predicted relative habitat suitability for Ornate Chorus Frogs across their distribution in the southeastern USA.

S4 Table. Number of Ornate Chorus Frog (*Pseudacris ornata*) records, observed between 2010 and 2024 with < 100 m estimated location error, used in our model to predict environmental suitability.

| State | Number of observations^a^ | Number of observations after spatial thinning^b^ |
| --- | --- | --- |
| North Carolina | 5 | 4 |
| South Carolina | 141 | 82 |
| Georgia | 222 | 60 |
| Florida | 491 | 251 |
| Alabama | 6 | 6 |
| Mississippi | 0 | 0 |
| Louisiana | 0 | 0 |
| Total | 865 | 403 |

^a^ This record set has been cleaned to exclude duplicates (i.e., identical coordinates), observations outside of the historical distribution, observations where the estimate of spatial error is missing, observations with a spatial error > 100 m, and observations prior to 2010.

^b^ We removed records that were within 120 m of another record.

S5 Table. Pearson correlation coefficient (lower) and Spearman’s rank correlation coefficient (upper) among environmental variables^a^ used in our MaxEnt model for Ornate Chorus Frogs (*Pseudacris ornata*) across their distribution in the southeastern USA. All correlations were less than |0.42|.

|  | Coniferous forest | Mixed forest | Wetland | Sand | Shrub-scrub forest |
| --- | --- | --- | --- | --- | --- |
| Coniferous forest |  | 0.326 | 0.085 | 0.307 | 0.411 |
| Mixed forest | 0.125 |  | -0.064 | -0.092 | 0.265 |
| Wetland | 0.011 | -0.138 |  | 0.313 | 0.121 |
| Sand | 0.315 | -0.029 | 0.224 |  | 0.307 |
| Shrub-scrub forest | 0.108 | 0.037 | -0.003 | 0.207 |  |

^a^All variables were continuous and each 30-m pixel in a raster represented the average value within a 450-m window.

S6 Table. Variable percent contribution and variable permutation importance for observation datasets thinned at 120 m and at 500 m.

| Variable | Thinned observations (120 m) | | Thinned observations (500 m) | |
| --- | --- | --- | --- | --- |
|  | Variable percent contribution | Variable permutation importance | Variable percent contribution | Variable permutation importance |
| Sand | 39.5 | 54.4 | 40.2 | 48.3 |
| Coniferous forest | 27.3 | 23.6 | 27.4 | 25.6 |
| Wetland | 16.1 | 6.5 | 15.7 | 9.4 |
| Mixed forest | 15.5 | 12.3 | 13.1 | 11.4 |
| Shrub-scrub forest | 1.7 | 3.2 | 3.6 | 5.3 |


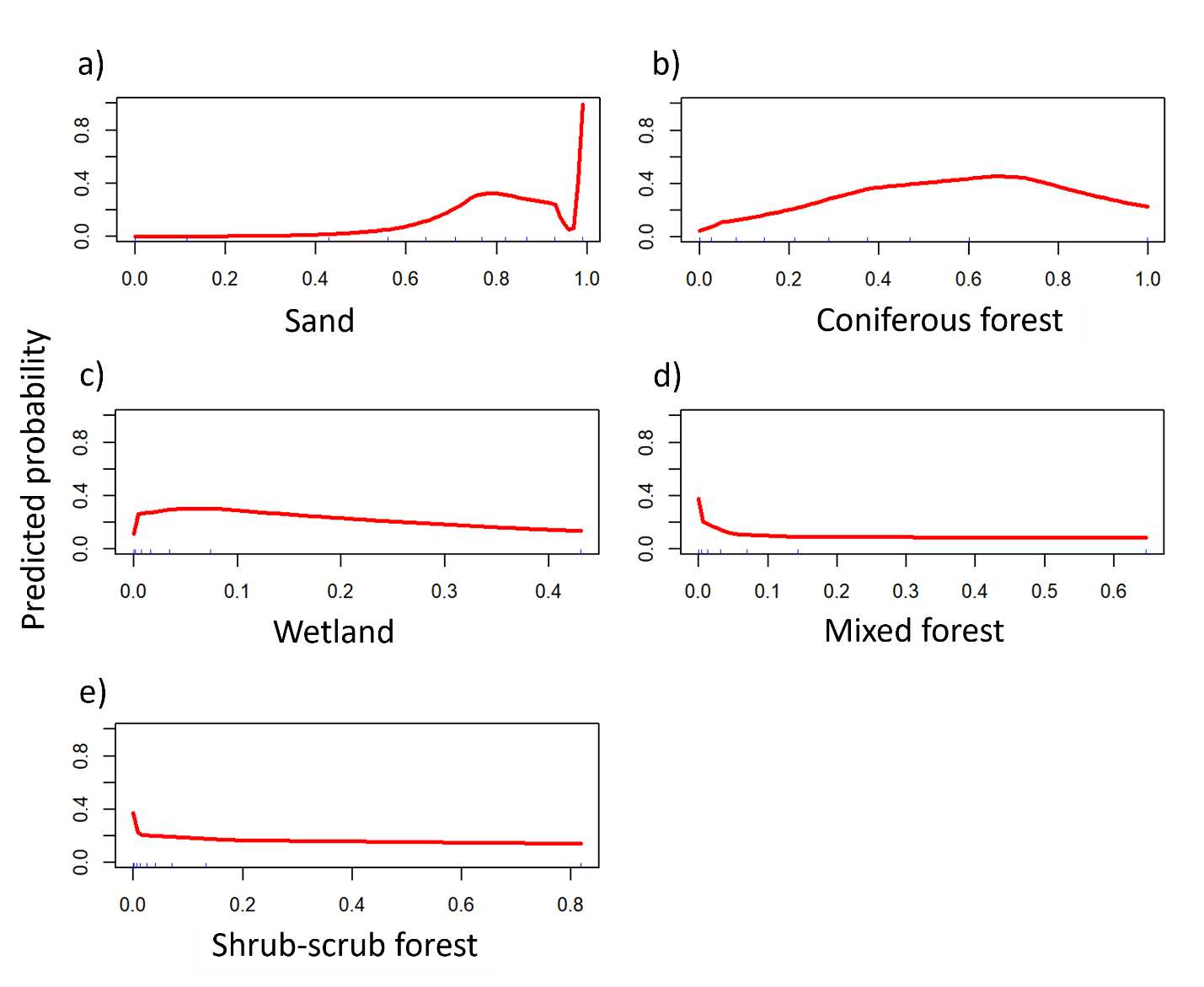


S2 Figure. Response curves of a) percent sand within the top 25-cm horizon, as a proportion within a 450-m radius; b) the proportion of coniferous forest within a 450 m-radius; c) the proportion of a 450-m radius that is ponds or wetlands < 20 ha in area; and d) and e) the proportion of mixed forest and shrub-scrub forest, respectively, in a 450-m radius, for the dataset with observations thinned at 120 m. Curves show how the predicted probability of presence changes as each variable is varied, with all other variables at their mean value. X-axes vary continuously from 0 to 1 and represent the mean value of each variable within a 450-m window, as described in the methods above.


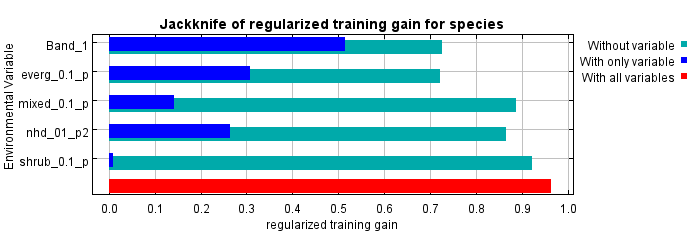


S3 Figure. Jackknife test of variable importance. The variables with the highest gain (percent sand within the top 25-cm horizon, as a proportion within a 450-m radius and the proportion of coniferous forest within a 450 m-radius) have the most useful information when used in isolation (dark blue bars). Those same two variables decrease the gain the most when omitted and appear to have the most information that is not present in the other variables (light blue bars). Plot produced using dismo version 1.3-16 (Hijmans et al. 2023) and Maxent version 3.4.3 (Phillips et al. [Internet]).


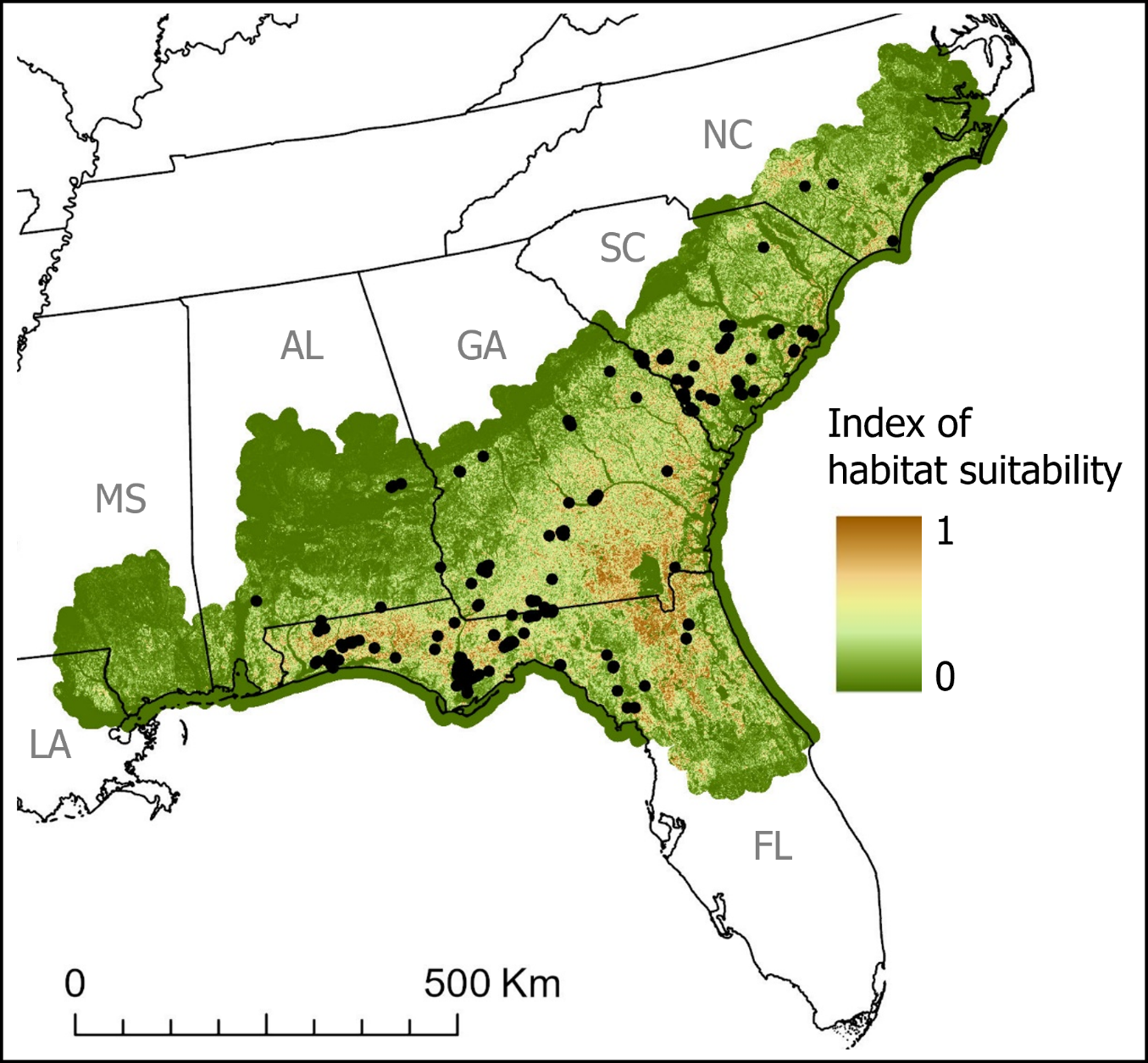


S4 Figure. Relative habitat suitability (cloglog) for Ornate Chorus Frogs (*Pseudacris ornata*) in the southeastern USA, and 403 observation records (thinned at 120 m; black dots), where brown represents relatively high suitability and green represents relatively low suitability. The outer polygon represents the Gap Analysis Project Species Range Map for *P. ornata* with a 10-km-wide buffer (USGS Gap Analysis Project 2018; CC0 1.0). North America basemap (CC BY 4.0) is from the Commission for Environmental Cooperation (2022).

We thank Stephanie Martinez for sharing the raster representing percent sand within the top 25-cm horizon. Any use of trade, firm, or product names is for descriptive purposes only and does not imply endorsement by the U.S. Government.

**References**

Aiello-Lammens, M. E., Boria, R. A., Radosavljevic, A., Vilela, B. and Anderson, R. P. (2015). spThin: an R package for spatial thinning of species occurrence records for use in ecological niche models. Ecography, 38: 541–545. URL https://onlinelibrary.wiley.com/doi/10.1111/ecog.01132.

Brown, L. E., & Means, D. B. (1984). Fossorial behavior and ecology of the chorus frog *Pseudacris ornata*. Amphibia-Reptilia, 5(3–4), 261–273.

Burrow, A. K. (2022). Conservation action plan: ornate chorus frog (*Pseudacris ornata*). Southeast Partners in Amphibian and Reptile Conservation.

Caldwell, J. P. (1987). Demography and life history of two species of chorus frogs (Anura: Hylidae) in South Carolina. Copeia, 114–127.

Commission for Environmental Cooperation (CEC). (2022). North American Atlas - Political Boundaries. Statistics Canada, United States Census Bureau, Instituto Nacional de Estadistica y Geografia (INEGI). Ed. 3.0. Vector digital data [1:10,000,000].

Dewitz, J., 2023, National Land Cover Database (NLCD) 2021 Products: U.S. Geological Survey data release, https://doi.org/10.5066/P9JZ7AO3.

Enge, K. M., Farmer, A. L., Mays, J. D., Castellon, T. D., Hill, E. P., & Moler, P. E. (2014). Survey of winter-breeding amphibian species. Final report. Florida Fish and Wildlife Conservation Commission. Fish and Wildlife Research Institute, Wildlife Research Section, Gainesville, 136.

Ethier, J. P., Fayard, A., Soroye, P., Choi, D., Mazerolle, M. J., & Trudeau, V. L. (2021). Life history traits and reproductive ecology of North American chorus frogs of the genus *Pseudacris* (Hylidae). Frontiers in Zoology, 18(1), 40.

Hijmans RJ, Phillips S, Leathwick J, Elith J (2023). dismo: Species Distribution Modeling. R package version 1.3-14, <https://CRAN.R-project.org/package=dismo>.

Kass, J. M., Muscarella, R., Galante, P. J., Bohl, C. L., Pinilla-Buitrago, G. E., Boria, R. A., Soley-Guardia, M., & Anderson, R. P. (2021). ENMeval 2.0: Redesigned for customizable and reproducible modeling of species’ niches and distributions. Methods in Ecology and Evolution. 12(9), 1602–1608. https://doi.org/10.1111/2041-210X.13628

Merow, C., Smith, M. J., & Silander Jr, J. A. (2013). A practical guide to MaxEnt for modeling species' distributions: what it does, and why inputs and settings matter. Ecography, 36(10), 1058–1069.

Phillips, S. J., Anderson, R. P., & Schapire, R. E. (2006). Maximum entropy modeling of species geographic distributions. Ecological Modelling, 190(3–4), 231–259.

Phillips, S. J., Anderson, R. P., Dudík, M., Schapire, R. E., & Blair, M. E. (2017). Opening the black box: An open‐source release of Maxent. Ecography, 40(7), 887–893.

Phillips, S. J., & Dudík, M. (2008). Modeling of species distributions with Maxent: new extensions and a comprehensive evaluation. Ecography, 31(2), 161–175.

Phillips, S. J., Dudík, M., and Schapire, R. E. [Internet] Maxent software for modeling species niches and distributions (Version 3.4.1). Available from url: http://biodiversityinformatics.amnh.org/open_source/maxent/. Accessed on 2024-7-25.

Soil Survey Staff. (2023). Gridded Soil Survey Geographic (gSSURGO) database for the conterminous United States. United States Department of Agriculture, Natural Resources Conservation Service. Available online at https://www.nrcs.usda.gov/resources/data-and-reports/gridded-soil-survey-geographic-gssurgo-database. (202310 official release).

R Core Team. (2024). R: A Language and Environment for Statistical Computing. R Foundation for Statistical Computing, Vienna, Austria. <https://www.R-project.org/>.

USGS. (2023). National Hydrography Dataset. Available at https://www.usgs.gov/national-hydrography/national-hydrography-dataset

USGS Gap Analysis Project. (2018). Ornate Chorus Frog (*Pseudacris ornata*) aOCFRx_CONUS_2001v1 Range Map: U.S. Geological Survey data release, https://doi.org/10.5066/F7DJ5DRK.

Zurell, D., Franklin, J., König, C., Bouchet, P. J., Dormann, C. F., Elith, J., Gusman, G. F., Feng, X., Guillera-Arroita, G., Guisan, A., Lahoz-Monfort, J. J., Leitão, P. J., Park, D. S., Peterson, T., Rapacciuolo, G., Schmatz, D. R., Schröder, B., Serra-Diaz, J. M., Thuiller, W., Yates, K. L., Zimmermann, N. E. & Merow, C. (2020). A standard protocol for reporting species distribution models. Ecography, 43(9), 1261–1277. DOI: 10.1111/ecog.04960.
