# Supplementary material for: Persistence of a declining anuran species across its distribution
Source: PLoS One. 2025 Sep 22;20(9):e0332991. doi: 10.1371/journal.pone.0332991 (PMC12453189; doi:10.1371/journal.pone.0332991)
Supplement: S1 File — S1 Appendix. Sources of Ornate Chorus Frog observation records. S2 Appendix. Examples of three buffer sizes to delineate Ornate Chorus Frog populations and sensitivity of persistence models to buffer size. S3 Appendix. Using the North American Amphibian Monitoring Program database to guide selection of non-target species to be used as an index of search effort. S4 Appendix. Predicting environmental suitability for Ornate Chorus Frogs using MaxEnt. S5 Appendix. Number of species detections per year (1900–2024) and relationship between observation date and persistence probability. S6 Appendix. Impact of predictor variables on probability of persistence. (ZIP) [file pone.0332991.s001.zip › supporting_information_R1_clean/S5_Appendix.docx]

Supplementary information supporting:

Persistence of a declining anuran species across its distribution

Erin L. Koen^1^, E. Hance Ellington^2,3^, William J. Barichivich^4^, Howard Kochman^4^, Kevin M. Enge^5^, and Susan C. Walls^4^

^1^ Cherokee Nation System Solutions, contracted to, U.S. Geological Survey, Wetland and Aquatic Research Center, Gainesville, Florida, USA, ^2^ Range Cattle Research and Education Center, University of Florida, Ona, Florida, USA, ^3^ Department of Wildlife Ecology and Conservation, University of Florida, Gainesville, Florida, USA, ^4^ U.S. Geological Survey, Wetland and Aquatic Research Center, Gainesville, Florida, USA, ^5^ Fish and Wildlife Research Institute, Florida Fish and Wildlife Conservation Commission, Gainesville, Florida, United States of America

# S5 Appendix. Number of species detections per year (1900–2024) and relationship between observation date and persistence probability.


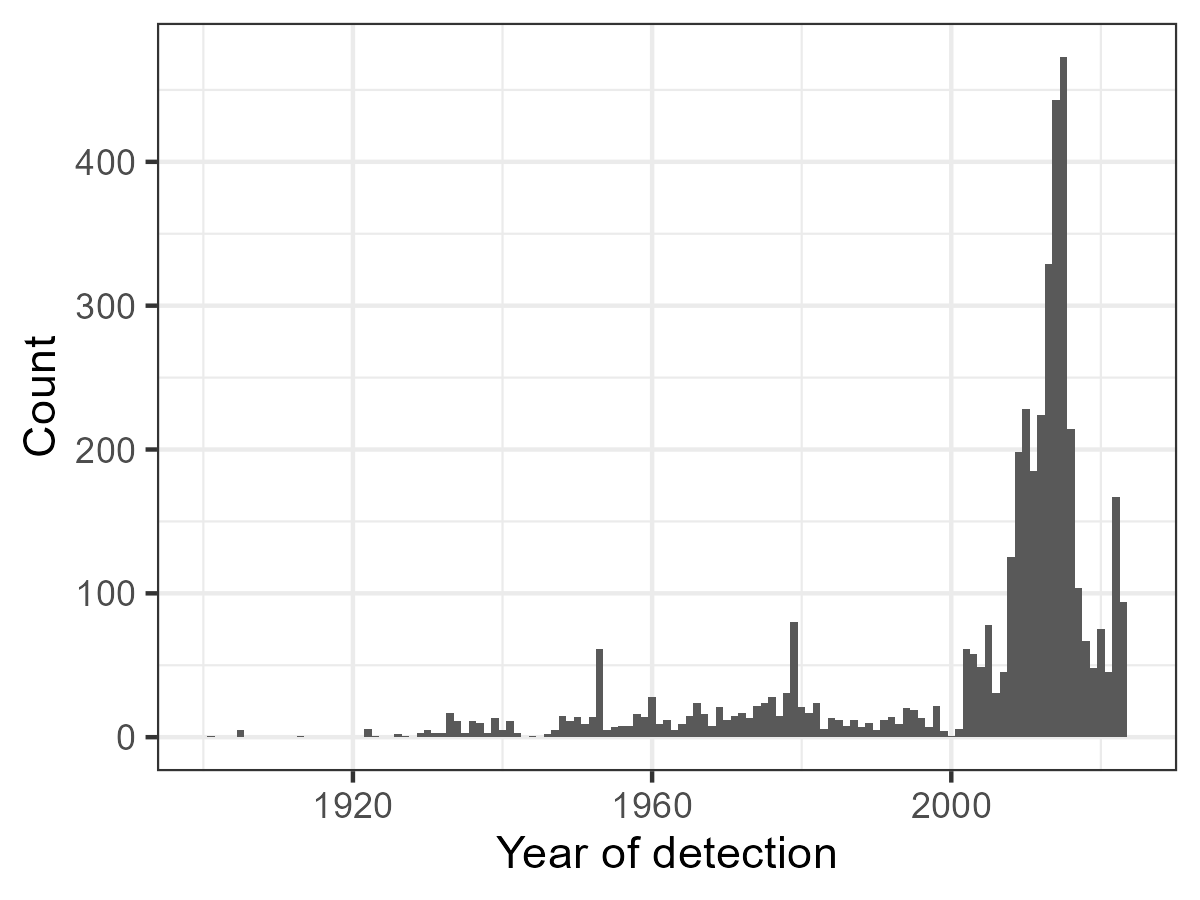


S5 Figure. Histogram showing the number of detections (non-target species pooled with Ornate Chorus Frog observations) per year from 1900 to 2024. Bin size is one year. The number of recorded and georeferenced observations per year increased over time, likely a reflection of technological changes through time, such as handheld GPS recording devices and community science databases. Refer to S3 Appendix for a list of the non-target species.


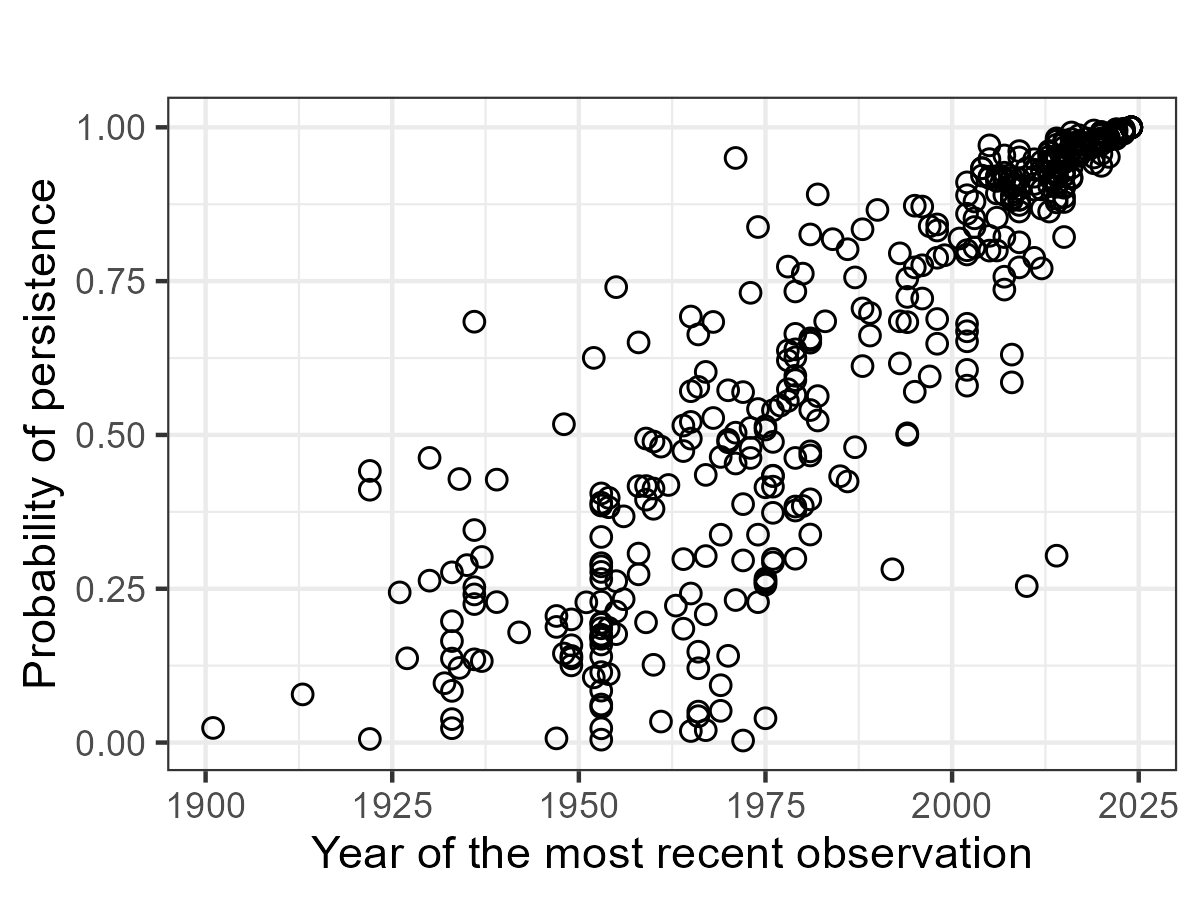


S6 Figure. Correlation between date of the most recently observed Ornate Chorus Frog within a population polygon and the probability of persistence. Pearson *r* = 0.88.
