# Supplementary material for: Persistence of a declining anuran species across its distribution
Source: PLoS One. 2025 Sep 22;20(9):e0332991. doi: 10.1371/journal.pone.0332991 (PMC12453189; doi:10.1371/journal.pone.0332991)
Supplement: S1 File — S1 Appendix. Sources of Ornate Chorus Frog observation records. S2 Appendix. Examples of three buffer sizes to delineate Ornate Chorus Frog populations and sensitivity of persistence models to buffer size. S3 Appendix. Using the North American Amphibian Monitoring Program database to guide selection of non-target species to be used as an index of search effort. S4 Appendix. Predicting environmental suitability for Ornate Chorus Frogs using MaxEnt. S5 Appendix. Number of species detections per year (1900–2024) and relationship between observation date and persistence probability. S6 Appendix. Impact of predictor variables on probability of persistence. (ZIP) [file pone.0332991.s001.zip › supporting_information_R1_clean/S6_Appendix.docx]

Supplementary information supporting:

Persistence of a declining anuran species across its distribution

Erin L. Koen^1^, E. Hance Ellington^2,3^, William J. Barichivich^4^, Howard Kochman^4^, Kevin M. Enge^5^, and Susan C. Walls^4^

^1^ Cherokee Nation System Solutions, contracted to, U.S. Geological Survey, Wetland and Aquatic Research Center, Gainesville, Florida, USA, ^2^ Range Cattle Research and Education Center, University of Florida, Ona, Florida, USA, ^3^ Department of Wildlife Ecology and Conservation, University of Florida, Gainesville, Florida, USA, ^4^ U.S. Geological Survey, Wetland and Aquatic Research Center, Gainesville, Florida, USA, ^5^ Fish and Wildlife Research Institute, Florida Fish and Wildlife Conservation Commission, Gainesville, Florida, United States of America

# S6 Appendix. Impact of predictor variables on probability of persistence


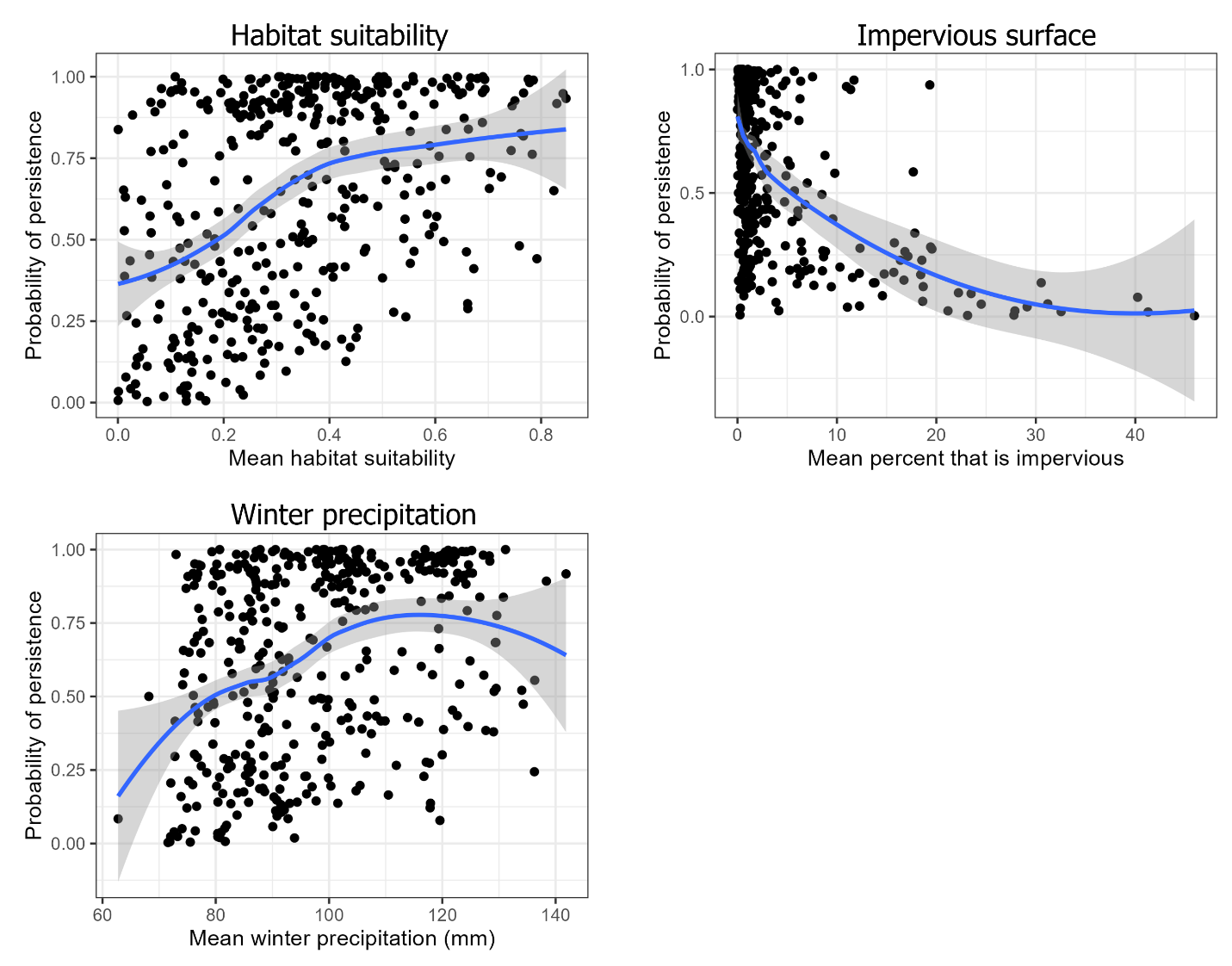


S7 Figure. Relationship between three predictor variables (described in section 2.7) on the probability of persistence of Ornate Chorus Frog (*Pseudacris ornata*) populations in 2024. Plots show individual Ornate Chorus Frog populations (black points; *n* = 407), Loess curves (blue lines), and standard error of the Loess curves (grey ribbons).


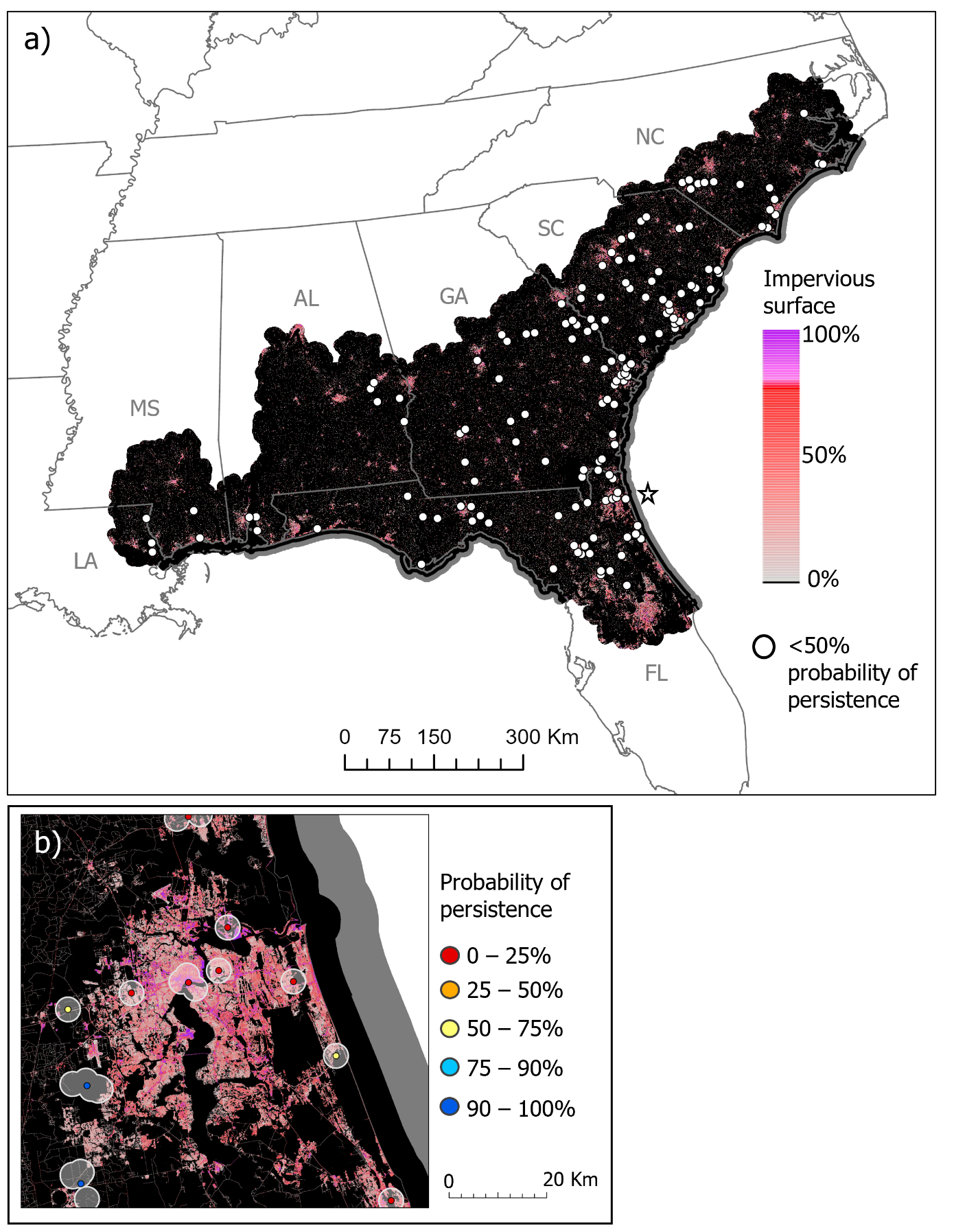


S8 Figure. The distribution of Ornate Chorus Frog (*Pseudacris ornata*) populations with a < 50% probability of persisting in 2024 in relation to areas with a high proportion of impervious surface; populations with > 50% probability of persistence are not shown in panel a. The star in panel a corresponds to the city of Jacksonville, Florida, which is enlarged in panel b. In panel b, all Ornate Chorus Frog populations in the Jacksonville region are shown (regardless of persistence probability; points represent population polygon centroids), with white polygons denoting population boundaries defined by overlapping 2.5-km radius buffers around Ornate Chorus Frog observations (individual observations not shown). The North America basemap (CC BY 4.0) is from the Commission for Environmental Cooperation (2022). The impervious surface map (Dewitz 2023) is in the public domain.

Commission for Environmental Cooperation (CEC). (2022). North American Atlas - Political Boundaries. Statistics Canada, United States Census Bureau, Instituto Nacional de Estadistica y Geografia (INEGI). Ed. 3.0. Vector digital data [1:10,000,000].

Dewitz, J. (2023). National Land Cover Database (NLCD) 2021 Products: U.S. Geological Survey data release, [h](https://doi.org/10.5066/P9JZ7AO3h)ttps://doi.org/10.5066/P9JZ7AO3.

S7 Table. Mean value of predictor variables within Ornate Chorus Frog (*Pseudacris ornata*) population polygons that are located in protected area or outside of a protected area.

|  | In a protected area | Not in a protected area |
| --- | --- | --- |
| Number of population polygons | 161 | 246 |
| Mean (SD) habitat suitability within polygons | 0.39 (0.19) | 0.31 (0.19) |
| Mean (SD) winter rainfall within polygons (mm) | 98.6 (16.3) | 96.7 (16.1) |
| Mean (SD) percent of a polygon that is impervious | 1.42 (2.07) | 4.95 (7.97) |
| Mean (SD) probability of persistence | 0.76 (0.27) | 0.55 (0.32) |
